# Global research on Unit 731 medical atrocities: from the 1950s to the 2020s

**DOI:** 10.1017/mdh.2026.10062

**Published:** 2026-10

**Authors:** Yan-jun Yang, Alissa Wang

**Affiliations:** 1 School of Humanities, Shanghai Jiao Tong University, Shanghai, China; 2 Independent Researcher, Toronto, Canada

**Keywords:** Unit 731, Second World War, medical atrocities, biological warfare, war crimes, human experimentation, historical understanding, war memory

## Abstract

During World War II, the Japanese Kwantung Army’s Unit 731 secretly conducted large-scale, inhumane, and unethical human experimentation in China, culminating in one of the most heinous medical atrocities, crimes against humanity, and war crimes in the Asia-Pacific theatre of the war. Despite the gravity of the subject matter and its historical significance, research on Unit 731 has, for a long time, been rather limited, but has gradually increased in recent decades. In this article, we identify several important characteristics and trends in research activities related to Unit 731, with a view to providing a general overview of the existing scholarship and to providing recommendations for future research.

From the 1950s to the 1970s, academic research started to accumulate. The second stage was one of rapid development from the 1980s to the 1990s. During this period, research efforts in Japan had a far-reaching influence and began to spread to China, Europe, and the U.S. Scholars from scientific, medical, and educational communities began participating in Unit 731 research successively. The third stage of multi-dimensional development began at the onset of the twenty-first century. Research expanded into comparative inquiries of Japanese medical atrocities and their Nazi counterpart; at the same time, research on many topics in diverse disciplines was also deepened and intensified. In addition to focusing on pragmatic issues such as reflections and actions of modern society and reconciliation, researchers have also been concerned with historical writing and collective memory.

## Introduction

The medical atrocities committed by the covert biological warfare research Unit 731^1^ of the Japanese Army are not only an important part of East Asian history, but also an indispensable event in the history of the Second World War, in global medical history, and in global history writ large. The history of Unit 731 is a classic case of the degeneration of medicine in the context of war, and one that is particularly important for scholars, politicians, and global-minded citizens alike to remember, record, understand, and reflect upon. Yet for the considerable period of 80 years after the war, the world outside of China and Japan was relatively unaware of the medical atrocities committed by Unit 731. This marks a stark contrast with the war memories, historical records, and social awareness associated with investigative research on Nazi medical atrocities. In 2025, which marks the 100th anniversary of the signing of the Geneva Protocol to the International Convention on the Prohibition of Chemical and Biological Weapons and the 80th anniversary of the end of the Second World War, the Chinese movie ‘731’ was released in countries including the United States, Canada, and Australia, promoting a nascent understanding of this important segment of history to English-speaking countries. Nevertheless, its limited popular appeal reveals not only a lack of general awareness of this segment of history among Western countries but also points to the importance of paving a more robust foundation for global historical understanding through rigorous academic scholarship.^2^

Since the 1950s, scholars around the world have conducted a notable amount of investigation and research into issues surrounding Unit 731. Research topics include the details of the Unit’s medical atrocities, the conduct of biological warfare, its post-war impact, issues of criminal responsibility, and contemporary reflections. Unit 731 research has been conducted at both macro and micro levels. On the macro level, scholars have firmly defined the Unit as the core Japanese organisation that researched and conducted biological warfare during the Second World War, as well as the headquarters for human experimentation. At the micro level, scholars have uncovered useful details such as the persons involved in the Unit, the organisation of the Unit, the types of medical experiments conducted for biological warfare, and details on its cover-up under epidemic prevention and water purification. All of this information contributed to the establishment of a more complete narrative of the process of Japanese biological warfare during the Second World War from planning to implementation. Over time, research perspectives have become increasingly diversified, expanding from traditional disciplinary research to interdisciplinary research. Theories and research methods from fields such as history, medical history, ethics, archaeology, biology, and international law have cross-fertilised. At the same time, geographical and historical boundaries in research are gradually being broken down. Research timelines are extending both forward and backwards, and the spatial scope of research is expanding from local and regional histories to global history. Throughout this expansion process, the excavation and organisation of archives and documents have been ongoing. There have also been new advancements in the analysis and verification of historical evidence related to the pre-war, wartime, and post-war periods. Overall, multi-dimensional research from the perspectives of the history of science and technology, medical history, and ethics has made significant breakthroughs.

This article has two key objectives. First, we provide an overview of the status, characteristics, and trends of research on Unit 731. Specifically, we present this general and introductory analysis of the state of the field for scholars in the English-speaking world, and we call upon international scholars of medical history to pay attention to this topic. Second, we provide an analysis of the key shortcomings of the existing scholarship and make a call for collaborative academic research between scholars of different nationalities to draw on respective strengths, fill important gaps in knowledge, and enrich research perspectives. Ultimately, we hope that this article provides a foundation for this academic collaboration and advancement.

## The beginning of Unit 731 research: lack of clarity and deliberate manipulation (1950s to 1970s)

After the end of the Second World War, the United States led the occupation and social transformation of Japan, and Japan began to transition from a ‘militaristic country’ to a ‘democratic country’.^3^ With the outbreak of the Korean War, the Cold War began to erupt between the United States and the Soviet Union, and ideological conflicts between the East and the West emerged one after another. It was in this post-war environment that the military doctors and officers of the former Unit 731 took on important positions in various sectors of Japanese society, including in their positions as university professors and presidents, practitioners of medicine, presidents of medical associations, corporate presidents, etc. Coming from the war era to the postwar era, many of these individuals decided to turn their backs on this history, and the medical community often adopted a general attitude of avoidance. As a result, silence towards these topics became the mainstream position in much of Japan’s postwar society.^4^ This general attitude lasted for the first 30 years of the post-war period, leading to the a lack of clarity surrounding the history of Unit 731.

However, despite this general context, a small number of research advances were made, most directly because of the publication of legal proceedings associated with the Khabarovsk trials,^5^ the military tribunal established by the Soviet Union from 25-29 December 1949, which tried twelve Japanese biological warfare criminals, including former members of Unit 731 and Unit 100.^6^ Thus, the period from the 1950s to the 1970s also marked the first stage of investigation and research efforts into Unit 731. These research advances were led by several exceptional Japanese scholars, civic organisations, journalists, former members of Unit 731, and others who knew about or were associated with the Unit. In contrast, the medical profession was generally not involved in these research efforts. This gave rise to the phenomenon that research on Unit 731 was confined to certain specific and isolated disciplines. In terms of content, research during this time focused on the wartime situation of Unit 731 and details of biological warfare and human experimentation. This resulted in the publication of a number of documentary novels, oral testimonies, and memoirs. Generally speaking, this period illustrated several distinctive characteristics. Research focused on the excavation of historical evidence and the initial stage of textual interpretation, and was aided by the production of relevant historical literature and the dissemination efforts of popular media. Although discussion on the topic of Unit 731 human experimentation had begun to surface, academic research was still in the period of genesis and Unit 731 had not yet become a well-known or well-defined area of study. However, the continuous emergence of a large amount of textual and oral evidence in this period sewed the seeds for the next stage of academic research and social understanding.

### Documentary works: experience, testimony, and memory

In 1950, the USSR made public the historical evidence concerning biological warfare in the Second World War for the first time through the publication of *Materials on the Trial of Former Servicemen of the Japanese Army Charged with Manufacturing and Employing Bacteriological Weapons.* ^7^ This publication was translated into different languages including English, Chinese, Russian, German, Japanese, and Korean. The *Materials on the Trial of Former Servicemen of the Japanese Army* includes indictments, confessions of the accused, witness statements, documentary evidence, and legal judgments. The historical facts of Unit 731’s human experimentation and its planning and deployment of biological warfare became known to the outside world for the first time, paving the groundwork for scholars to begin historical investigation and research.

The medical crimes committed by Unit 731 first entered the field of vision of Japanese medical historians in 1956. Only scattered written records existed at that time, and systematic academic investigation had yet to take shape. Research was still in its early stages, characterised by a lack of comprehensive knowledge. For instance, *Special Forces Unit 731* published in 1956 was the first official publication written by a former Unit 731 member.^8^ Human experimentation, biological warfare, and the Khabarovsk War Crimes Trials were presented in oral history form from the perspective of the author Akiyama Hiroshi as a personal witness.^9^ Then, in 1957 Masao Tamura wrote in *A Century of Japanese Medical History* that ‘among the direct combat activities supported by Japanese physicians, the most inhumane and horrifying were undoubtedly the preparations for biological warfare… This unit produced bacteria, employed biological weapons, and conducted human experimentation’.^10^ The 1967 publication, *History of Science and Technology in Japan,* included a section on Military Medicine, providing an initial overview of the Army Medical School’s research into biological and chemical warfare, and excerpting trial records from the Khabarovsk Trials in the Soviet Union. This work states explicitly that ‘under the guise of the Kwantung Army’s Epidemic Prevention and Water Supply Department, Shiro Ishii, Director of the Army Medical School’s Epidemic Prevention Research Laboratory, initiated the development and experimentation of biological weapons. Although all facilities were destroyed and documents burned upon Japan’s defeat, the detailed circumstances remain unclear. The trials records from the Soviet Khabarovsk Trials constitute the sole available source for understanding this general process.’^11^ This demonstrates that due to limitations in historical records and the silence of most individuals associated with Unit 731, the truth about the unit long remained shrouded in mystery.

In 1966, Yoshinaga Reiko published *The Horror of Human Experimentation: Memoirs of a Kwantung Army Female Dependant*, in which she, as a firsthand witness and dependant of a Kwantung Army member, recounted the atrocities of human experimentation.^12^ This was followed by Shimamura Takashi’s publication of *Three Thousand Human Experiments* in 1967.^13^ The works of Yoshinaga and Shimamura relied mostly on the *Materials on the Trial of Former Servicemen of the Japanese Army* as the major reference material. These publications mainly recorded the wartime situation of Unit 731, and introduced the topics of wartime human experimentation, the biological warfare attacks in Zhegan and Changde, and historical evidence of the related Unit 100. These works contributed to raising Japanese society’s awareness of Unit 731 and of the Khabarovsk War Crimes Trials in particular. However, publications on Unit 731 in this period were reflective of the perceptual understanding of the war experience of the witnesses. As they were mainly based on oral testimonies and supplemented to a certain extent by textual materials, these publications should be categorised as documentary literature, rather than rigorous and critical academic research.

The 1967 publication, *History of the University of Tokyo Faculty of Medicine: A Centennial Review* recounts the faculty’s wartime experiences, including the shortening of academic programs and the establishment of affiliated medical departments. It documents particularly the US B-29 bombings of Tokyo and the atomic bombings of Hiroshima and Nagasaki, detailing the faculty’s responses to arduous wartime conditions.^14^ Throughout the entire text of the *Centennial History*, there is no mention of the Faculty of Medicine’s active participation in the war or its aiding of the war effort, nor does it address the issues of war crimes or war responsibility. Instead, the *Centennial History* adopts a victim’s perspective to denounce the atrocities committed by the US military, while avoiding any mention of the Faculty’s own active involvement in the war effort. This omission perhaps reflects a broad ambivalence towards the historical role of the Faculty of Medicine in the Japanese war effort.^15^

### Subject transformation: from perceptual understanding to rational research

Takasugi Shingo’s *The Original Sin of Japanese Medicine: Human Experimentation and Wartime Responsibility* and *Human Experimentation in Japan: Its Ideology and Structure*, both published in 1973 were representative academic achievements of the 1970s.^16^ Takasugi’s research made two main breakthroughs: first, he placed Unit 731 in the context of medical ideology, medical education, and the wartime Japanese medical system, integrating the theories and methodologies from the discipline of the social history of medicine, and analysing how science was mobilised under state power and in line with state policies under the wartime system;^17^ second, from the perspective of war responsibility, he explored the ‘ideology of objectification of human beings’ in wartime Japanese medical education, defining the ‘original sin of Japanese medicine’ as ‘serving the rulers while oppressing the people who were ruled’.^18^ These two works published by Takasugi can be seen as a watershed between popular reading and academic research. Before Takasugi’s publication, publications related to Unit 731 had dabbled in a certain degree of academic scholarship, but they remained at the level of combing and interpreting the evidence from the Khabarovsk War Crimes Trials. However, after Takasugi’s research publications, mainstream publications gradually increased in academic rigour, venturing into the disciplines of political history, history of science and technology, history of medicine, and ethics. Overall, works on Unit 731 were no longer relying dominantly on oral historical materials but instead focused on the integration of oral testimonies with archival materials and emphasised the excavation, examination, and analysis of historical evidence.

At the same time, a large number of veterans’ memoirs and witnesses’ oral testimonies continued to be published during this period. In 1977, Tomokatsu Matsumura published the *Memoirs of the Vice Chief of Staff of the Kwantung Army* in 1977, which included the section ‘Lieutenant General Ishii, the authority on biological warfare’, and documented Ishii Shiro’s wartime activities as a firsthand witness.^19^ In 1979, Banseisha published in Tokyo *Documents: Biological Warfare*, which included new historical materials such as the ‘Membership Directory of the *Seikonkai*’, marking the first time that the organisation of Unit 731 and its post-war activities were made known to the public.^20^

Based on the publications from the 1950s to 1970s, several key characteristics emerge. First, the authors of the memoirs and documentaries were those who were directly involved in the activities of Unit 731 or who had close relationships with the members of the Unit. In contrast, the Japanese medical profession paid little attention to issues related to Unit 731, which is likely due to the fact that many doctors associated with Unit 731 still occupied important positions after the Second World War,^21^ and they unambiguously chose to remain silent with regards to this dark past.^22^ Second, most publications focused on revealing the existence and wartime activities of Unit 731 through roughly outlining the basic structure of the Unit, detailing the human experimentation and biological warfare atrocities committed by the Unit, and preliminarily analysing the internal and external causes of such inhumane behaviour. Third, those who had personal involvement in or close relationships with Unit 731 were most active in areas such as media interviews, testimony gatherings, and publishing books. Thus, many of the publications appeared in the form of documentary works and personal memoirs. Although a large number of oral testimonies, photographic evidence, drawings, and figures were cited, the investigation and use of original archival materials were not yet common. Hence, this period paved the way for scholars to enhance the scope of archival research.^23^ Fourth, the influence of publications of this period was largely confined to Japan. Academia in China, Europe, and the United States paid little attention to the issue of Unit 731 prior to the 1980s.

## Growth and spread to Europe and the United States: the social dissemination of historical narrative and the reflective activities of the international community (1980s–1990s)

In the 1980s and 1990s, Japanese society once again experienced a counter current of denial of histories of imperial aggression, with events such as public denials of the Nanjing Massacre, high-level visits to the Yasukuni Shrine, and ongoing controversies over the content of school history textbooks, all of which had a significant impact on the historical understanding of the Japanese people.^24^ At the same time, there were also civic movements in Japanese society to hold to account those responsible for war crimes. These large-scale civic activities, often organised around slogans such as ‘peace’, ‘anti-war’, and ‘anti-nuclear’, also attracted a large amount of popular attention through media promotion, social activities, touring exhibitions, and publications.^25^

This period coincided with the rise of anti-war forces in Japan, given the emergence of a wide number of social movements, particularly peace movements, throughout Japanese society.^26^ Investigation, dissemination, and publication related to Unit 731 flourished across a variety of genres and forms, including thematic studies, documentary compilations, documentary literature, oral histories, and television documentaries. During this period, there were several prominent lawsuits, most importantly Ienaga Saburo’s series of textbook lawsuits (1965–97) and the biological warfare lawsuit filed by 180 Chinese victims of Unit 731 on 11 August 1997, with the assistance of Japanese lawyers, at the Tokyo District Court.^27^ With the dissemination by news media, the support of civil society groups, and the impact of touring exhibitions, these lawsuits played an important role in pushing forward Unit 731 research and had a large impact not only on Japanese society but also on China, Europe, and the United States. With the continuous excavation of public and private archives in Japan, the United States, and Russia, newly released evidence enhanced enthusiasm for academic studies on Unit 731.^28^ As a result, publications on Unit 731 in the 1980s and 1990s experienced a transformation from being shaped predominantly by the authors’ personal experiences and perceptions to being influenced by more rational and impartial discussions, and from predominantly literary narrative styles to historiographic research.

### The excavation of textual materials: systematic study from the perspective of the history of science and technology

Building on the excavation of trial materials, oral histories, memoirs, and wartime publications, Japanese scholars such as Tsuneishi Keiichi began to turn their attention to public archives in Japan, the United States, and Russia. *The Vanished Germ Warfare Unit* ^29^ and *Organized Crime by Medical Professionals* ^30^ published by Tsuneishi Keiichi had a significant impact on Unit 731 research in this period. Tsuneishi utilised a large amount of archival materials, including *Fifty-Year History of the Army Medical School*, *Journal of the Army Medical Corps*, *Secret Records - Nagata Tetsuzan*, *Hygienic History of Army in the Great East Asian War* and *Report on the Study of Epidemic Prevention at the Army Medical School.* ^31^ Tsuneishi analysed the establishment and expansion of Unit 731 from the perspective of the history of science and technology. Tsuneishi’s educational background in physics had a direct influence on his focus on a microscopic examination of scientific and technological history, and his extensive use and systematic analysis of archival materials provided a comprehensive account of the organisation, scope, and biological warfare and human experimentation activities of Unit 731.

Tsuneishi conducted multiple visits to the U.S. National Archives in Fort Detrick and Dugway Proving Ground for on-site research and was the first to discover the U.S. collection of archival materials on Japanese biological warfare. He then selected about five hundred pages from the archives and translated them into Japanese, and included the translated text in the book *Target Ishii: Espionage by Unit 731 and the U.S. Army.* ^32^ In 1994, Tsuneishi wrote *Organized Crime by Medical Professionals* based on the newly discovered *Epidemic Prevention Research Report of the Army Medical School.* ^33^ Compared to previous works, the authenticity, accuracy, and reliability of *Organized Crimes by Medical Professionals* was greatly improved.^34^ In this period, studies on Unit 731 entered the realm of sophisticated academic studies, incorporating elements such as standardised verification, fact-checking, and the integration of history and theory.

### The driving force of literature and history: *The Devil’s Gluttony* and its implications

In 1981, well-known Japanese writer Morimura Seiichi’s book *The Devil’s Gluttony* ^35^ gained popularity in Japan. From 19 July to 3 October 1981, the work was serialized in the Japanese Communist Party’s newspaper *Shimbun Akahata*. It was later published as a three-volume book, becoming a bestseller that underwent multiple revisions and reprints. Over three million copies were sold in Japan, and the work was translated into Chinese and Russian to be sold overseas. As a result, the history of Unit 731 caught the public’s attention and had a tremendous impact on Japanese society. At the same time, studies on Unit 731 drew the continued attention of the academic community, and an increasing number of Japanese scholars became involved in this scholarly discussion. This marked the beginning of systematic research on Unit 731. After *The Devil’s Gluttony* gained popularity in Japan, Morimura and Ikebe Shinichiro, two well-known Japanese musicians, established the Devil’s Gluttony Chorus. Over the following decades, the Chorus performed in hundreds of performances across Asia, Europe, and the United States on the themes of anti-war, peace, and humanitarianism, which further increased awareness of Unit 731 around the world. Morimura Seiichi continued to publish and re-print a series of books, including *The Devil’s Gluttony*, *Proof of Man*, *New Proof of Man*,^36^ and *Unit 731 on Trial*,^37^ leading to a significant impact on the publishing industry as well as growing concerns within Japanese society. For example, Shimamura Takashi’s *3,000 Human Experiments: The Crimes of the Japanese Army’s Germ Warfare Unit* ^38^ was revised and reprinted, and Ishida’s *The Devilish Japanese Military Doctor* ^39^ and Hiyama Yoshiaki’s documentary novel *Pursuit of the Biological Warfare Unit Doctor* ^40^ were published to a great reception in Japanese society. The profit motive of the Japanese publishing industry further promoted the publication of works on topics related to Unit 731. During this period, memoirs of witnesses and people who were associated with Unit 731 were published one after another. These include Army Surgeon Lieutenant colonel Ryoichi Naito’s *A Collection of Ryoichi Naito’s Miscellaneous Writings* in 1980,^41^ Yoshikai Natsuko’s *Unforgettable Memory: A Record of the Vivisection of Dr. Yuasa, a Military Doctor* ^42^ in 1981, Gunji Yoko’s *Testimony: Unit 731 and the Ishii Detachment* ^43^ and *Uncovering the Truth: Ishii’s Germ Warfare Unit* ^44^ in 1982, Akimoto Sueo’s *Questioning the Logic of Medicine: Based on Experiences in Unit 731* ^45^ in 1983, Koshi Sadao (from transportation team)’s *The Rising Sun Is Dyed Red with Tears: Confessions of a Member of Unit 731* ^46^ in 1983, Yoshimura Hisato’s (leader of frostbite research team) *Looking Back on My 77th Year* ^47^ in 1984, Takezawa Masao’s (member from water purification department) Tōgō Society Journal^48^ and 731 research society’s *Biological Warfare Unit* ^49^ in 1986.

Outside of Japan, Britain was the first European country that became concerned about the history of Unit 731. British authors Robert Harris and Jeremy Paxman cowrote *A Higher Form of Killing: The Secret Story of Chemical and Biological Warfare*,^50^ which was first published in English in 1982, and then in Chinese by Qun Zhong Publishing in 1988. A British independent television production company produced a documentary *Unit 731: Did the Emperor know?* in August 1985.^51^ The two producers of the documentary, Peter Williams and David Wallace, published a book titled *Unit 731: Japan’s Secret Biological Warfare in World War II* ^52^ four years later. The book became a representative work of European and American academia in the period and remains one of the most cited works in the English language on this topic.^53^ Scholars from the US also published several influential works on the issue of Unit 731. For example, John Powell published an article ‘A hidden chapter in history’ in the *Bulletin of the Atomic Scientists* ^54^ in October 1981 and this article revealed for the first time the secret US-Japanese deal on biological warfare. Sheldon Harris from the Department of History of California State University published an article named ‘Japanese Biological Warfare Experiments and Other Atrocities in Manchuria, 1932-1945, and the Subsequent United States Cover Up: A Preliminary Assessment’.^55^ Harris then conducted extensive fieldwork in China and Japan, which allowed him to finish the manuscript of *Factories of Death: Japanese Biological Warfare: 1932-45, and the American Cover-up* ^56^ in 1994. Harris’s book had influential status in European and American academia and was later translated into Japanese and Chinese.^57^

During this period, there were several dissertations by graduate students from universities in the U.S. on the topic of Unit 731. For example, Walter Eugene Grunden dedicated a chapter in his dissertation to discuss chemical and biological warfare using sociological research methodology in *Science under the Rising Sun: Weapon Development and the Organization of Scientific Research in World War II Japan.* ^58^ The dissertation analysed the unique characteristics of Unit 731 from the perspective of the vertical social structure and traditional norms of behaviour in wartime Japan. In addition to the use of American materials, Japanese scholar Ota Masakatsu published *Unit 731’s Immunity: Secret Files of the Biological Warfare Unit* in 1999.^59^ The book utilised the oral testimonies of Lieutenant Colonel Niizuma Seiichi and abundant material from the collections, which allowed the author to comprehend the process of Niizuma’s secret liaison and negotiation with Kitano Masaji and Masuda Tomosada in conjunction with the US military forces.

### The lawsuit of Ienaga: the ‘push’ of local movements

In the 1980s and 1990s, the textbook lawsuits of Ienaga Saburo^60^ created an uproar in the Japanese government and in civil society. When reviewing Japan’s high school history textbooks, the Japanese Minister of Education proposed a revision to ‘completely delete’ all content on Unit 731.^61^ In the end, the Supreme Court of Japan rejected the proposal of the Minister of Education. The social impact generated by this case was not contained to the issue of the textbook litigation itself. News and public opinion concerning the court’s judgment broadened the case’s impact within and beyond Japan, and promoted Japanese society’s further recognition of the criminal history of Unit 731.^62^

The important roles in ‘historical investigation’ and ‘academic solidarity’ during the Ienaga lawsuit played by Morimura Seiichi and Matsumura Takao were not widely known by the public.^63^ Importantly, the Supreme Court of Japan referred to the ‘results of historical research’ to reach a judgement on Ienaga’s lawsuit. As a result, the research, investigation, and narration of Morimura and Matsumura provided strong historical evidence for the lawsuit.^64^ Morimura’s *Unit 731 on Trial* explained the motivation, process, and conclusion of the investigation of the historical facts of Unit 731, and Morimura Seiichi’s Witness Statement was submitted to the Court as supplementary evidence. Importantly, *Unit 731 on Trial* also included the witness statements of Hata Ikuhiko and Eguchi Keiichi.^65^ Matsumura Takao utilised 168 pages to record ‘Opinions of Matsumura Takao’ and ‘Testimony of Matsumura Takao’ in *Arguments for Unit 731.* The work cited archival evidence in Japanese, English, and Russian, and in particular, it presented official direct historical sources kept by Japan’s Ministry of Foreign Affairs and Ministry of Defense. Matsumura also gave a detailed account of the origins, contents, and values of his edited work *Documents Created by Unit 731.* ^66^ Morimura and Matsumura introduced, commented on, and refuted the content of the textbook regarding Unit 731, compiled many historical facts, summarised the state of research, and drew objective conclusions, which had a wide social effect as the lawsuit continued to fester and had a direct impact on the dissemination and promotion of the historical facts of the Unit 731 in Japan and beyond.^67^

Furthermore, two large-scale lawsuits against Japan were initiated by Chinese civil society groups with strong support from well-known lawyers in 1995 and 1997.^68^ From 1995 to 2007, the two lawsuits were heard by the Tokyo District Court and the Tokyo High Court, which confirmed the facts of human experimentation victimisation and the facts of the crime of biological warfare, but both rejected the claims from the plaintiffs.^69^ During this period, legal experts, historians, sociologists, and the domestic and international media continued to pay attention to this event. The rapid intervention of media and its real-time reporting also drew a high degree of attention from Japanese society to this case. In particular, the simultaneous appearance of the Japanese legal team, victims, perpetrators, and third-party witnesses in court directed influenced the restoration of historical facts of bacteriological warfare, the determination of historical authenticity, and the final verdict of the Tokyo court.^70^ At the same time, Chinese and Japanese academic communities cooperated in the editing of the eight-volume *Biological Warfare on Trial.* ^71^ *Biological Warfare on Trial* recorded relevant materials concerning evidence collection, lawsuits, and judgements, thus effectively promoting international understanding of the history of Unit 731, human experimentation, biological warfare, and the US-Japanese deal, laying the groundwork for a wider range of investigations and research.

To summarise, research on the issue of Unit 731 during this second period was characterised by the following features: first, while Japanese academia still dominated research in the field, and while research findings were mainly published in Japanese, its influence was no longer confined to Japan, but had spread through translation to China, Europe, and the United States. Second, the 1980s marked the pioneering stage of academic research, and the 1990s marked a stage of rapid development, with the participation of scholars in the fields of science and technology, medicine, and education. Third, in contrast to the previous period, when the Japanese people were ‘fighting alone’, research groups were established, including the Japan Audit Bureau of Circulations, the Society for Disclosure of Historical Facts on Biological Warfare of the Japanese Army, the NPO Corporation Unit 731 Biological Warfare Resource Center, and the Society for the Study of the Problems of the Discovery of Human Bones on the Ruins of Military Medical Schools, among others. Lastly, academic research made great progress with the continuous excavation of English- and Japanese-language archival documents, and civil movements within Chinese and Japanese society promoted continuous in-depth investigations and research into Unit 731. At the same time, Japanese scholars gradually shifted their focus from unilateral investigation from the perspective of the victimising party to focusing on evidence collection that relied on the victimised party.

## East-West interaction and the rise of pluralism: the stratification of historical understanding and different memories across regions and countries (the twenty-first century and beyond)

After half a century of historical excavation and the accumulation of academic studies, alongside the natural generational changes of the Japanese medical community, the bond between the post-war medical community and the wartime medical community gradually began to break down, and many medical professionals began to directly participate in research on Unit 731. Some of them graduated from Faculty of Medicine, Kyoto University and Kanazawa University, while others had taught at the Faculty of Medicine at Tohoku University, Shiga Medical University, and Fukushima Prefectural Medical University, among others.^72^ Due to the genuine concern of Japanese medical professionals about the history of Unit 731, an extensive amount of investigative research was produced during this period, which robustly promoted research on the scientific and medical aspects of this issue. It can be said that the extensive involvement of the Japanese medical community made it possible for the issue of Unit 731 to enter the field of East Asian medical history and, possibly in the near future, to become part of global medical history.

Research on Unit 731 conducted by international scholars has also become more mature and sophisticated since the dawn of the twenty-first century. Specifically, Unit 731 research demonstrated a three-dimensional development marked by the diversification of research perspectives, internationalisation of research horizons, and globalisation of the scope of research. Research on Unit 731 has developed both horizontally through the rise of comparative research and vertically through the deepening of research on existing topics, leading to the systematisation of macro-research and refinement of micro-research. Research methods were constantly innovated as well: the transition from single discipline to interdisciplinary research was completed during this period, with the utilisation of theories and methods from disciplines such as history, medical history, international jurisprudence, ethics, and archaeology. Representative achievements of Japanese academia in this period include *Verification of Human Experimentation: Unit 731 and Nazi Medicine* ^73^ by Omata Waichiro, *War and Medicine* ^74^ by Nishiyama Katsuo, the academic papers and investigation reports published in the *Journal of Research Society for Fifteen Years War and Japanese Medical Science and Service*, journalist Nishizato Fuyoko’s *Unit 731: The Japanese Army*’*s Biological Warfare Unit That America Pardoned*,^75^ and Aoki Fukiko’s *731: Unveiling the Darkness of Unit 731 and Shiro Ishii.* ^76^ Key academic achievements in Europe and the US include Nie Jingbao and Guo Nanyan’s *Japan’s Wartime Medical Atrocities: Comparative Inquiries in Science, History and Ethics*,^77^ Jeanne Guillemin’s *Hidden Atrocities: Japanese Germ Warfare and American Obstruction of Justice at the Tokyo Trial*,^78^ and Kenneth L. Port’s *Deciphering the History of Japanese War Atrocities: The Story of Doctor and General Shiro Ishii.* ^79^

### In-depth study by Japanese scholars from the perspective of medical history and ethics

At the beginning of the twenty-first century, Japanese scholars made important breakthroughs in terms of collecting, organising, and publishing historical materials. Major research outcomes include *A Collection of Documents on Unit 731’s Biological Warfare* ^80^ and *Report on Epidemic Prevention Research at the Army Medical School (Part 2).* ^81^ Building on the discovery of wartime Japanese historical materials such as the *Imoto Journal and the Kaneko Junichi Theses*, these two books have achieved a breakthrough in confirming the basic historical details such as the time and place of Japan’s biological weapon attack, the mode of operation, and the number of victims of biological warfare.

Precisely due to the Japanese medical community’s sustained focus on studying Unit 731 from the perspective of medical history and ethics, studies of Unit 731 and its relationship with issues of war, medicine, and ethics increased in depth and comprehensiveness. For example, Omata’s *Verification of Human Experimentation: Unit 731 and Nazi Medicine* examined Germany as a comparator for the study of wartime Japanese doctors. Omata compared the similarities and differences between Japan and Germany before, during, and after the war in terms of historiography and medical historiography, historical understanding and ethical judgement, personal and organisational relationships, and law and medicine.^82^

With the establishment of the Research Society for 15 Years War and Japanese Medical Science and Service, hundreds of Japanese medical practitioners, clinicians, and ethicists were involved in discussions regarding Unit 731. This brought forward profound changes to the breadth of investigation and research on Unit 731. Japanese scholars investigated, researched, and promoted an understanding of the medical crimes committed by Unit 731 doctors, and have published more than four hundred papers and investigation reports on topics such as biological warfare, the history of medicine, human experimentation, responsibility for war, and ethical reflection.^83^ Japanese scholars have made new progress in horizontal expansion by venturing into comparative research in two areas. First, they began comparing the similarities and differences between the wartime medical practices of Japan’s Unit 731 and Nazi medical atrocities. They conducted this comparative analysis from a variety of perspectives, including through the lenses of nationalism, racism, organisational structures, budgetary systems, and medical systems.^84^ Second, they began to compare the historical narratives, discourse, and reflective practices of post-war Japan and post-war Germany on the issue of medical crimes, in order to elucidate the historical memories, ethical reflections, and contemporary values in different spaces and environments.^85^

In 2013, Nishiyama published *War and Medicine* within the framework of the Fifteen Years’ War and the Japanese medical field.^86^ The book examined the relationship between the War of Aggression against China and organisations in Japan’s medical community such as the Japanese Industrial Hygiene Society, the Japanese Health Society, and the Japanese Medical Association, revealing the war responsibilities and post-war perceptions of the these groups. In 2014 and 2016, Nishiyama and the other scholars edited two influential collections: *NO MORE 731: The Japanese Army’s Biological Warfare Unit – A Conscientious Investigation by Medical Professionals* ^87^ and *War and Unit 731, Universities and Medical University* .^88^ In these works, Nishiyama selected eighteen articles from thirteen authors, including nine medical doctors, and examined medical education and the medical system in the context of fifteen years of war, focusing on issues such as wartime mobilisation, the ‘military-academic commonwealth’, war coordination, and post-war responsibilities at the Tohoku Imperial University School of Medicine, Kanazawa Medical University, Kyoto Imperial University, Tokyo Imperial University, and Kyushu Imperial University.

More recently, Tsuneishi Keiichi, in his book *Complete History of Unit 731: The Ishii Institution and the Military Academic-Industrial Government Complex*,^89^ not only reviewed the entire process of the establishment, expansion, and demise of Unit 731, but also referred to historical materials such as *The Diary of Imoto Kumao*, *The Report of Kaneko Junichi*, and the doctoral dissertations of Kyoto University to uncover the plague-bacteria warfare operations of the Unit 731 at the specific locations of Nong’an, Quzhou, Ningbo, Changde, Guangxin, Guangfeng, and Yushan. In April 2022, *Unit 731 and the University* ^90^ edited by Professor of Clinical Medicine at Kyoto University and former director of Kyoto Central Hospital, Yoshinaka Takeshi, was published by Kyoto University Press in Japan. The book is divided into two parts: the first is the Japanese translation *of Unit 731: The Devil’s Laboratory – Auschwitz in the East* (see section 3.3), and the second part consists of eleven essays written by eleven Japanese scholars, including Yamamuro Shinichi, Mitsuyama Masao, Tsuchiya Takashi, and Minami Norio. This work became one of the more influential academic works of recent years.

### Overseas encounters: research and ethical perspectives

Since the founding of ‘the Research Society for 15 Years War and Japanese Medical Science and Service’ on the initiative of Japanese physicians in 2000, an increasing number of Japanese doctors and medical historians have directly participated in investigating the historical evidence of Unit 731. The intensifying participation of the Japanese medical community further drew the attention of the European and American bioethics academic bioethicists to the issue of Unit 731. Since 2000, scholars from the United States, the United Kingdom, Russia, Belgium, and other countries have published nearly 100 articles on Unit 731 in international journals of medicine and bioethics, including the *American Journal of Bioethics*, *Asian and International Journal of Bioethics*, and *The Lancet.* Scholars mostly discussed the issue from the perspective of bioethics and engaged in in-depth academic dialogues on the occurrence, harm, impact, and response to medical crimes in wartime Japan. For example, in 2006 overseas Chinese scholar, Jingbao Nie of New Zealand, published an article ‘The US Cover-up of Japanese Wartime Medical Crimes: Complicity in the Name of National Interest and Two Suggestions for Contemporary Action’^91^ in *The American Journal of Bioethics*. Nie’s article clearly laid out the historical facts surrounding the history of the US cover-up, including its motivations, its global moral significance, and concrete proposals for steps forward. This article revealed that the United States secretly traded human experimentation intelligence with Unit 731 after the war while simultaneously covering up Unit 731’s war crimes. Based on Nie’s article, the *American Journal of Bioethics* subsequently published six discussion articles at the same time, and a relatively intense academic debate began: some scholars believed that the US bioethics committees and the US government should respond as soon as possible and sincerely apologise; other scholars believed that it may be more valuable to prompt the United States to reveal the facts and underlying causes of Japanese and Nazi medical crimes in Japan so that others can learn from these ethical lessons and historical experiences. Jingbao Nie’s article recommended that ‘the US government issue a formal apology, making it clear that it now condemns the Japanese war crimes including those associated with BW activities and human experimentation, and offers compensation in some form to redress its own actions in covering up these atrocities’.^92^ Katerin Devolder in her article ‘U.S. Complicity and Japan’s Wartime Medical Atrocities: Time for a Response’ stated that ‘Perhaps a statement, instead of an apology (like in the Guatemala case), would be an appropriate response, as well as directing the Bioethics Commission to write a brief report on the U.S. involvement in the coverup of the Japanese medical experiments’.^93^ Julie Aultman in the article ‘When Saying Sorry Is Not Enough: Acknowledging Past Wrongs in Human Subjects Research’ concluded that ‘a public apology may carry little meaning compared to alternative actions such as acknowledging the unethical Japanese experiments and the victims of these crimes, and ultimately preventing future faulty ethical reasoning and wartime exigency through enhanced critical thought, public discourse, ethics education and advisement, and responsiveness to global values and human rights’.^94^

Following this series of articles, Kenneth L. Port, a legal scholar who has studied in Japan for seven years and is fluent in Japanese and familiar with Japanese culture, approached Unit 731 research from a different perspective. Focusing on Ishii Shiro as a historical figure, Port’s book *Deciphering the History of Japanese War Atrocities: The Story of Doctor and General Shiro Ishii* ^95^ utilised American archives and on-site investigations to microscopically examine Ishii’s life from the perspective of his family background, schooling, military experience, secret dealings, and post-war life. Shortly afterwards, Jeanne Guillemin’s *Hidden Atrocities: Japanese Germ Warfare and American Obstruction of Justice at the Tokyo Trial* returned to the theme of the US cover-up. Rather than focusing on the examination of the criminal evidence of Unit 731, Guillemin’s work explored the sharing of information on biological warfare between the United States and the UK, the detailed process of the negotiation between the United States and Japan, the obstruction of justice at the Tokyo trial, and the transmutation between national interests, international rules, and medical ethics.^96^ Focusing more on the details of Unit 731 experimentation, through an examination of testimony from the Khabarovsk War Crime Trials, Canadian scholar Kishor Johnson in his 2022 article ‘A Scientific Method to the Madness of Unit 731’s Human Experimentation and Biological Warfare Program’ argued that Unit 731’s human experiments were scientifically rigorous by the standards of the time. He concluded that ‘Given the Japanese scientists’ wealth of background knowledge in microbiology, access to the most recent literature, and active participation in the broader scientific community, the investigators were well prepared to integrate the scientific method into their program of human experimentation’.^97^

More recently, Valerie J. Cranmer argued in the article ‘In Judgment of Unit 731: A Comparative Study of Medical War Crimes Trials after World War II’ that “the prosecution of the crimes of the Imperial Japanese Army’s Unit 731 are often compared to the prosecution of the crimes of the Nazi doctors. These comparisons emphasize immunity for the Japanese, whereas the Nazis were prosecuted for their actions. However, this comparison is an inaccurate one, since their composition, scope, and framework were different.”^98^ Cranmer contended that “the United States avoided prosecuting Unit 731 because they could not replicate the same clear legal framework used to successfully prosecute the Nazis. The Soviet Union recognized the strategic implications of the United States’ decision not to try Unit 731 and saw an opportunity to strike a moral blow, not only by convicting 12 Japanese military members at the Khabarovsk Trial.”^99^ The article also noted that Nazi Germany, Britain, the United States, and the Soviet Union all had programs that investigated the use of BW or chemical warfare weapons during World War II. However, Unit 731 is used as the prime example to describe the evils of BW. Analysis of Unit 731 rarely extends beyond the program’s existence and human experimentation in its research. Moreover, writers treat BW programs worldwide irrespective of one another and rarely analyse them as an aggregate to define a general global trend in the interwar period of states that developed and researched BW.^100^

### Chinese academia: from the periphery to centre stage

Following the litigation campaign against Japan, the discovery and disclosure of the ‘special transfer’ archives, and the opening-up of the archives of the United States and Japan, several Chinese institutions including Shanghai Jiao Tong University, the Exhibition Hall of Crime Evidence of Japanese Army Unit 731, the Harbin Academy of Social Sciences, and Hunan University of Arts and Science began to focus on Unit 731 research. Chinese scholars gradually utilised original archives, focusing not only on macro issues such as the preparation, implementation, and impact of human experimentation and biological warfare but also on the in-depth excavation, analysis, and interpretation of specific cases in localised contexts, while turning towards multi-dimensional research in history, archaeology, ethics, and the history of medicine.

Over the past 20 years, there have been two main types of outputs from Chinese academia: compilations of primary sources and academic analysis. In the field of evidence compilation, for example, the National Archives Administration of China published the *Selected Confessions of Japanese War Criminals Who Invaded China* (120 volumes),^101^ which included the confessions and written records of the former members from Unit 731, including Sakakibara Hideo and Hata Masayoshi. In 2015, the China Peace Publishing House published the *Factual Record of the Crimes of the Unit 731 of the Japanese Army in Invasion of China* (60 volumes), which included archival data on Japan’s biological warfare preserved by China, Japan, and the United States.^102^ The Japanese materials were collected from the Ministry of Defense of Japan, the National Public Library, and the National Library of Congress. As for the materials on the American side, they mainly came from the Library of Congress and Stanford University, as well as from Chinese official archives. This series has high historical and research value, and its compilation and publication have allowed Chinese scholars to surpass the comprehensiveness and scope of the previous compilation of historical materials on Unit 731 by Japanese scholars. In 2018, the China Social Sciences Press published a series of five books in the volume *Studies on the Biological Warfare Waged by the Japanese Invaders in Changde*,^103^ which mainly focused on biological warfare in Changde.

A distinctive characteristic of the investigation and research on Unit 731 of the Chinese academic community is the application of archaeological theory and methods to the field of medical history. *Archaeological Excavation Report of the Former Site of the Bacterial Laboratory and Special Prison of the Japanese Invading Army’s Unit 731* ^104^ contributed to the result of the creation of a new paradigm for the integration of historical and archaeological research. This work supplements, rectifies, reshapes and introduces a new research methodology to studies of Unit 731. This report was not entirely confined to the analysis of archaeological findings, but instead placed archaeological findings in a multidisciplinary context of documentary records, interviews with the historical witnesses, and oral histories. In addition, *Namelist of the Kwantung Army’s Epidemic Prevention and Water Supply Department*,^105^ *Namelist of the Unit 1855*,^106^ and *Namelist of the Southern Army’s Epidemic Prevention and Water Supply Department* ^107^ by Yang Yan-jun; *Information on Japan’s Biological Weapons Operational Survey* (6 volumes) by Wang Xuan and Kondo Shoji; and *Biological Warfare Archives of the Japanese Invasion Forces* by the National Library of China Publishing House were published in recent years. While the content of the Chinese archives has some overlap with the US archives, the two archival collections also differ in scope and textual organisation.

In the field of academic research, historical excavation still dominates the study of Unit 731, and research outcomes under the perspectives of medical history, ethics, and archaeology continue to emerge. Some major publications include *Japan’s Biological Warfare Against China* ^108^ by Zhiyuan Chen, *Research Report on the Crimes of Japanese Biological Warfare Against China* ^109^ by Zhonghou Xie, and *A History of Biological Warfare* ^110^ by Jianxin Zhu. These works documented the existence, wartime behaviour, war responsibility, war damage, and post-war impact of forces engaged in biological warfare. With the confirmation of these macro-issues, a number of micro-issues in the field of the history of biological warfare have become the subject of new research perspectives, as scholarly attention shifted from the study of Unit 731’s global significance to the detailed analysis of specific cases. There were some major research papers, such as ‘The Main Content, Historical Value, and Significance of Newly Discovered Documents on Japanese Biological Warfare’^111^ by Xiliang Wang, ‘Unit 731 and the Allied POW Camp in Shenyang’^112^ by Gao Jian, and ‘Examining the Personnel Composition of Unit 731’^113^ by Yanjun Yang. In particular, Yang’s ‘Examining the Personnel Composition of Unit 731’ relies on the article ‘Namelist of the Kwantung Army’s Epidemic Prevention and Water Supply Department’ and uses relevant historical materials of the Japan Ministry of Defense and the National Archives of Japan to clarify major historical issues such as the composition of Unit 731, its personnel composition, source of personnel, and its overall troop size, leading to the promotion of further related research.

A significant advance by Chinese academics came from the publication of Tam Yuehim and Yang Yanjun’s English-language book, titled *Unit 731: Laboratory of the Devil, Auschwitz of the East* ^114^ in 2018. It is one of the most comprehensive English-language scholarly works published overseas by Chinese scholars, which utilised materials from the US National Archives analyse the detailed process of the US military’s investigation into Unit 731 and record the traumatic memories of the families of victims who had suffered from human experimentation, an issue that had been less explored in previous works.

Overall, research on Unit 731 in China has shown two distinctive features. First, Chinese research tended to cross geographical and disciplinary barriers of research. With the deepening of research and active academic exchanges between regions, Chinese scholars were no longer confined to the scope of ‘local history’ but were gradually paying attention to the study of the preparation, implementation, and impact of biological warfare from a global perspective. Scholars from archaeology, medical history, and cultural heritage have also participated in the scholarly discussion on the history of biological warfare, which has led to the organic integration of research institutes with specialised institutions for site survey and archaeological excavation and display.

Second, Chinese research is starting to tend towards developing increasingly standardised, rigorous, and scientific methodology and analysis. For a long time, due to the constraints of pragmatic factors such as the difficulty of collecting historical data and the lack of research funds, Chinese scholars were not able to make much use of the core archives and did not adequately identify relevant historical data, leading to a failure to distinguish between the damage caused by epidemics that resulted from biological warfare and epidemics from natural causes. As a result, in academic research, there have been a few cases in which epidemic prevention records have been used as historical materials of biological warfare, leading to a mistaken recognition of people killed by natural epidemics as victims of biological warfare, and as a result, leading to an overestimation of the number of people killed and injured by biological warfare.^115^ With the improvement of research conditions and the gradual enhancement of levels of research, a number of Chinese academics have strengthened the excavation and utilisation of foreign archives, improved the accuracy of empirical research, improved the process of argumentation, and drawn historical conclusions in a more cautious and evidence-based manner.

In addition, Korean scholars have published more than ten research papers in Korean journals on the topic of Unit 731, such as ‘Bare Life in Manchuria and the Victims of Human Experiments in the Special Prison of Unit 731’^116^ by Suh Yi-Jong from Seoul National University and ‘The Memory of Biological Warfare and New Directions for Patriotic Education Base Construction in China’^117^ by Sihn Kyu-Hwan from Yonsei University. Russian scholars including Alepko have published more than 20 papers in Russian journals, including *The Khabarovsk War Crimes Trial of 1949 and the Cruel Human Experiments of Japan’s Unit 731.* ^118^

To conclude, research on Unit 731 since the twenty-first century exhibits several key characteristics. First, the extensive involvement of the Japanese medical community and the academic interest from European and American bioethics scholars led mainstream research on Unit 731 during this period to be dominated by observations and analyses from the perspectives of medical history and ethics. Thus, there were both horizontal comparative studies of wartime Japan, post-war Japan, and Nazi Germany and post-war Germany, as well as vertical in-depth studies within different disciplines. Second, Japanese scholars utilised the Research Society for War and Medicine^119^ as their main academic platform, focusing on the experience of war, and the fate of individuals from the perspectives of medical history, military medicine, and sociology. These Japanese scholars published hundreds of papers in the fields of the history of war and medicine, medicine and ethics, and covering topics including the culpability of those involved in bacteriological wars, medical crimes, and civil lawsuits. Compared with the twentieth century, the number and quality of research have increased considerably. Third, Japanese scholars tended to overlook the research findings of Chinese scholars in the investigation and research on biological warfare crimes and were more inclined to pay attention to the works of European and American scholars. Finally, European, American, South Korean, and Russian scholars have also contributed actively to the academic discussion during this period, not only publishing nearly one hundred academic papers but also more than ten books related to Unit 731, illustrating the beginnings of a productive East-West academic interaction.

## Conclusion: reflection and future perspectives

Over the past 70 years, global academic research on Unit 731 has experienced notable development, both theoretically and methodologically. Research progressed from documentary literature, the documentation of war experience, and memoirs to interdisciplinary research in the fields of the history of science and technology, history of medical, ethics, political history, and international law. In terms of scope of research, new progress has been made in the horizontal direction with increasing amounts of comparative studies of Japan and Germany, and the expansion of research efforts from Japan to China, South Korea, Europe, and the United States. In the vertical dimension, research has deepened in a number of different ways, leading to the convergence on common concerns from scholars of both the East and the West.

Recently, Japanese academia has slowed down its pace of research, while research in Europe and the United States is becoming more mature and systematic. Judging from the research situation in the past 5 years, Japan has demonstrated a gradual decline in research enthusiasm. This can be attributed to the fact that the previous generation of Japanese scholars and social activists has grown older, and the new generation of Japanese society has not been actively reflecting on, responding to, or facing up to the history of the Second World War for a long time. In contrast, European and American scholars have published a large number of new research papers. They tend to focus less on the details of local and regional history and more on Unit 731 in the broader context of the history of the Second World War, the Cold War, and the post-Cold War era, the comparison between Japanese and Nazi medical atrocities, and the themes of reflection, collective memory, peace, and reconciliation.

Despite notable academic achievements globally over the past decades, progress has also been uneven and limited in several ways. While most research on Unit 731 has academic and practical value, there are still important shortcomings such as lack of historical data, insufficient research, and hasty conclusions. In addition, there are occasional contradictions in Unit 731 studies, which speaks to the importance of follow-up research. Overseas research also reminds the Chinese academic community of the need to focus on accuracy and objectivity in research into Unit 731, so as to enhance the discursive power and influence of historical narrative, text interpretation, and social communication.

Therefore, although progress has been made, there is still considerable room for improvement. In particular, there is room for further research on the historical understanding, post-war damage, post-war reflection, and ecological impact of Unit 731. It is especially important for scholars worldwide to recognise and seriously engage with the recent research findings of the Chinese academic community, which has generally been neglected by academic communities in Japan, Europe, and the United States. At the same time, it is also necessary for Chinese academics to reflect on the shortcomings of their research methodology and theoretical frameworks. This would provide the necessary foundation for much-needed collaborative research among scholars internationally. Only when there is an open interaction from a wide range of diverse perspectives can academics meaningfully improve historical understanding, historical memory, and reconciliation, and work towards the common goal of preventing the recurrence of historical tragedies. There is still a long way to go.

## Supporting information

10.1017/mdh.2026.10062.sm001 Yang and Wang supplementary material Yang and Wang supplementary material

## Footnotes

1 Unit 731, established in early 1933 and disbanded in 1945, was headquartered in Harbin, Northeast China, with five additional branches. During its existence, it secretly conducted large-scale human experiments and biological warfare research, resulting in the deaths of over 3,000 people from China, the United States, the Soviet Union, and Korea due to illegal human experimentation. Unit 731 has become synonymous with Japan’s wartime medical crimes.

2 Following the Second World War, Japan did not engage in profound reflection on its wartime culpability. Instead, it actively transformed its role by collaborating with the occupying forces, exhibiting a dual character: an eagerness to embrace the postwar era coupled with a deliberate turning away from its history. As American scholar John W. Dower wrote in *Embracing Defeat: Japan in the Aftermath of World War II*, the Emperor’s loyal subjects, who just yesterday had sworn allegiance to ‘one hundred million hearts as one’ and ‘one hundred million souls as one’, overnight transformed into advocates to ‘one hundred million collective repentances’ and ‘turning over a new leaf’.

3 For background on the US involvement in Japan’s postwar reconstruction, see Edwin O. Reischauer , The United States and Japan (Cambridge: Harvard University Press, 2014); Jennifer M. Miller , Cold War Democracy: The United States and Japan (Cambridge: Harvard University Press, 2019).

4 Keishirō Karita, `War and the Medical Community’, *Journal of Japanese Scientists*, 43, 2 (2008), 3; Maruyama Hiroshi, `Retrospect and prospect of the Japanese Society for hygiene’, in Japanese Society for Hygiene 50th Anniversary Editorial Committee (ed.), *Fifty-Year History of the Japanese Society for Hygiene* (Japanese Society for Hygiene, 1984), 557.

5 For background on the Khabarovsk Trials, see Valentyna Polunina , ‘The Khabarovsk trial: The Soviet riposte to the Tokyo Tribunal’, in Kirsten Sellars (ed.), Trials for International Crimes in Asia (Cambridge: Cambridge University Press, 2016); Valentyna Polunina , ‘From Tokyo to Khabarovsk: Soviet war crimes trials in Asia as Cold War battlefields’, in Kerstin von Lingen (ed.), War Crimes Trials in the Wake of Decolonization and Cold War in Asia, 1945–1956: Justice in Time of Turmoil (Cham: Springer International Publishing, 2016); James Larry Taulbee , ‘Khabarovsk war crime trial’, in James Larry Taulbee (ed.), War Crimes and Trials: A Primary Source Guide (Santa Barbara: ABC-CLIO, 2018).

6 Unit 100, formally known as the Kwantung Army’s Unit 100, was established in Changchun, China, in 1936. This unit was responsible for conducting research on biological weapons and carrying out unlawful human experiments. It was dissolved in 1945.

7
*Materials on the Trial of Former Servicemen of the Japanese Army Charged with Manufacturing and Employing Bacteriological Weapons* (Moscow: Foreign Languages Publishing House, 1950).

8 Hiroshi Akiyama, *Tokushu Butai 731* (Special Forces Unit 731), Kyoto: Sanichi Shobo, 1956.

9 Hiroshi Akiyama, *Tokushu Butai* *731(Special Forces Unit 731)* (Tokyo: Sanichi Shobo, 1956), 42–46, 90–95, 96–97.

10 Masao Tamura, *A Century of Japanese Medical History* (Tokyo: Nipponrinshosha Co., Ltd., 1957), page 236.

11 The History of Science Society of Japan, *History of Science and Technology in Japan*, Vol. 25 (Tokyo: Dai-ichi Hoki Co., Ltd., 1967), page 309.

12 Reiko Yoshinaga, *Jintai Jikken no Kyōfu: Aru Kantōgun Josi Gunzoku no Shuki* (The Horror of Human Experimentation: Memoirs of a Kwantung Army Female Dependent), Osaka: Naniwa Shobo, 1966.

13 Takashi Shimamura, *Sanzennin no Seitaijikken: Kantōgun Nazo no Saikin Himitsuheiki Kenkyūjo* (Three Thousand Human Experiments: The Mysterious Biological Weapons Research Institute of the Kwantung Army), Tokyo: Hara Shobo, 1967.

14 The Centennial Anniversary Society of the Faculty of Medicine, *History of the University of Tokyo* Faculty of Medicine: A Centennial Review (Tokyo: The University of Tokyo Press, 1967), 202–203, 206.

15 The Centennial Anniversary Society of the Faculty of Medicine, *History of the University of Tokyo Faculty of Medicine: A Centennial Review,* Tokyo: the University of Tokyo Press, 1967.

16 Shingo Takasugi, *Nihon Iryō no Genzai: Jintai Jikken to Sensō Sekinin* (The Original Sin of Japanese Medicine: Human Experimentation and Wartime Responsibility) (Aki Shobo, 1973). And Takasugi Shingo, *Nihon no Jintai Jikken: Sono Shisō to Kōzō* (Human Experimentation in Japan: Its Ideology and Structure) (Mikasa Shobo, 1973).

17
*Ibid.*, Shingo Takasugi, i.

18
*Ibid.*, Shingo Takasugi, v.

19 Tomokatsu Matsumura, *Kantōgun Sanbō Fukuchō no Syuki* (Memoirs of the Vice Chief of Staff of the Kwantung Army) (Fuyō Shobō, 1977).

20 Nichikan Kankei wo Kirokusuru Kai (the Association for Recording Japan-Korea Relations), *Shiryō “Saikin-sen”* (Documents: Biological Warfare) (Banseisha, 1979).

21 Yanjun Yang, *Unit 731: The Fall of Medicine* (Beijing: Zhonghua Book Company, 2025), 184–185.

22 It should be noted that after the Second World War, Japan’s medical community exploited every opportunity to conceal historical truths. They quietly shelved the history of Unit 731, maintaining silence while uniting to suppress the emergence of the truth and evading historical accountability at every turn. It also cannot be overlooked that during the war, institutions such as Tokyo Imperial University, Kyoto Imperial University, and Keio University supplied Unit 731 with large numbers of graduates at different times, including over 70 PhDs of medicine, science, agriculture, and pharmacy. These medically trained elites and graduates mostly became senior managers within Unit 731, forming the core force behind human experimentation and biological warfare research. After the war, universities such as the University of Tokyo, Kyoto University, and Keio University awarded doctoral degrees to over 60 former members of Unit 731, laying the academic and social foundations for them to occupy key positions in Japan’s medical community. Notably, more than 100 former Unit 731 members went on to serve as university presidents, deans of medical schools, medical association presidents, and university professors. Regarding their past criminal activities, they tacitly chose silence and evasion, leveraging their prominent positions within Japan’s medical community to obscure the historical truth of Unit 731’s medical crimes. For more information, see the following works: Katsuo Nishiyama, in *War and Medicine* (pp. 204–209), compiled statistics on Unit 731-related personnel who acquired doctoral degrees from Kyoto University. Yasushi Wakata noted in his article ‘Clarifying the Reality of the Pathology Department in Wartime’, published in *The Centennial History of the Department of Pathology, Kyoto University Faculty of Medicine* (Kyoto: The Publication Committee for the Centennial History of the Department of Pathology, Kyoto University Faculty of Medicine, 2008), 280–281, Shozo Azami, in his article ‘“The Medical Crime in the Fifteen Years’ War” and Our Task Today’, *Journal of 15-Years War and Japanese Medical Science and Service*, 8, 2, (2008), 28, all have noted Unit 731’s cover-up of historical facts, evasion of the past, and its personnel’s postwar employment.

23 During this period, archives related to Unit 731 preserved at Japan’s National Archives and the US National Archives were not effectively excavated or utilised. Consequently, investigations into the truth about Unit 731 remained confined to general historical narratives.

24 For background, see Ria Shibata , ‘Memories of the War and Japanese “Historical Amnesia”’, Education and Society, 35, 1 (2017), 5–25 ; Christopher Barnard , ‘Isolating Knowledge of the Unpleasant: The Rape of Nanking in Japanese high-school textbooks’, British Journal of Sociology of Education, 22, 4 (2001), 519–530 ; Daqing Yang , ‘Revisionism and the Nanjing Atrocity’, Critical Asian Studies, 43, 4 (2011), 625–648 .

25 See Glenn D. Hook , Militarisation and Demilitarisation in Contemporary Japan (London: Routledge, 2003); Mari Yamamoto , ‘Japan’s Grassroots Pacifism’, Japan Focus, 3, 2 (2005); Akiko Naono , ‘“Ban the Bomb! Redress the Damage!”: The History of the Contentious Politics of Atomic Bomb Sufferers in Japan’, Asian Journal of Peacebuilding, 6, 2 (2018), 223–246 ; Akihiro Ogawa , ‘The New Prominence of the Civil Sector in Japan’, in Theodore C. Bestor , Akiko Yamagata and Lyon Bestor Victoria (eds.), Routledge Handbook of Japanese Culture and Society (London: Routledge, 2011), 186–197 ; Jennifer Chan , Another Japan is Possible: New Social Movements and Global Citizenship Education (Stanford: Stanford University Press, 2008).

26 Peng Hu, ‘Japanese Social Movements and Its Characteristics in the Post-war Era’, *Japanese Studies*, 5 (2015), 98–119.

27 Importantly, on 27 August 2002, the Tokyo District Court issued a ruling acknowledging the facts of biological warfare atrocities but dismissed the plaintiffs’ claims for compensation. On the textbook lawsuits, see: Y. Nozaki, *War Memory, Nationalism and Education in Postwar Japan, 1945–2007: The Japanese History Textbook Controversy and Ienaga Saburo’s Court Challenges* (Palgrave Macmillan, 2008). On the biological warfare lawsuit, see: A. Ishii, ‘Saikin Sen Higaisha Sosho no Shiteki Imi’ [Historical Significance of the Biological Warfare Victims’ Lawsuit], *Japanese Journal of Contemporary History*, 8 (2003), 45–72.

28 National Archives of Japan. Digital Archive. https://www.digital.archives.go.jp (accessed May 29, 2026). U.S. National Archives and Records Administration. “Japanese War Crimes Records.” https://www.archives.gov/iwg/japanese-war-crimes (accessed May 29, 2026). Cunliffe William H., comp. Select Documents on Japanese War Crimes and Japanese Biological Warfare, 1934–2006. Washington, D.C.: National Archives and Records Administration, 2007. https://www.archives.gov/iwg/japanese-war-crimes/select-documents.pdf (accessed May 29, 2026). Russian State Archives. Khabarovsk Trial Records, 1949. Online archive, 2021. http://khabarovsk1949.rusarchives.ru (accessed May 29, 2026; note: access limited in some regions).

29 Keiichi Tsuneishi, *Kieta Saikinsen Butai: Kantōgun Dai 731 Butai* (The Vanished Germ Warfare Unit: The Kwantung Army’s Unit 731), Kaimeisha, 1981. This book is a seminal work by Keiichi Tsuneishi, who documented Unit 731 from the perspectives of scientific mobilisation and medical technology through the lens of the history of science and technology. In this volume, he focuses on tracing the key figures of Unit 731 – Shiro Ishii and Masatsugu Kitano – along with the circumstances surrounding medical mobilisation at the Army Medical School. The core themes remain the special transfers, human experimentation, and biological warfare. However, Koishi did not examine the Unit 731 site or utilise English-language archives. Consequently, while this work stands as a representative study on the subject, it reflects the limitations of available documentation at the time and lacks more systematic data support to some extent. Naturally, these issues were better addressed in Koishi’s subsequent works.

30 Keiichi Tsuneishi, *Igakushatachi no Soshiki Hanzai: Kantōgun Dai 731 Butai* (Organized Crime by Medical Professionals: Unit 731 of the Kwantung Army), Asahi Shimbun, 1994. Also Tsuneishi Keiichi and Tomizo Asano, *Saikinsen Butai to Jiketsusita Futari no Igakusha* (The Biological Warfare Unit and the Two Medical Doctors Who Committed Suicide), Shinchosha, 1982. And Tsuneishi Keiichi, *H*y*ōteki Ishii: Nanasanichi Butai to Beigun Chōhō Katsudō* (Target Ishii: Unit 731 and U.S. Intelligence Activities) (Otsuki Shoten, 1984). And Tsuneishi Keiichi: *Senjō no Ekigaku* (Epidemiology of the Battlefield) (Kaimeisha, 2005).

31 The aforementioned materials all constitute primary historical sources directly linked to Unit 731. Compared to oral testimonies, they possess higher levels of authenticity and accuracy, thereby holding significant value for academic research: The *Fifty-Year History of the Army Medical School* is the official 50-year chronicle of the Imperial Japanese Army Medical School (1886–1936), the institutional precursor to Unit 731. The *Journal of the Army Medical Corps*, the official publication of the Japanese Army Medical Corps, was published from its inaugural issue in March 1903 until its discontinuation in August 1945. Issued monthly, it produced a total of 376 volumes throughout its run, making it a highly representative military medical journal in modern Japan. Notably, members of Unit 731 published a substantial number of military medical research papers within its pages. The *Secret Records – Nagata Tetsuzan* is the definitive published collection of Nagata Tetsuzan’s classified papers, memoranda, letters, and internal military records (1929–1935), compiled by his associates and the ‘Nagata Tetsuzan Publication Society’. The *Hygienic History of Army in the Great East Asian War* is the official military historical document focusing on army military hygiene, epidemic prevention and medical deployment during the Pacific War and full-scale invasion of China. This work records military health management, disease prevention measures, troop sanitation work, medical service arrangements and field epidemic control practices implemented by the Japanese army across various battlefields during the Great East Asian War. The *Report on the Study of Epidemic Prevention at the Army Medical School* is the official, detailed records of research directly aimed at developing and deploying biological weapons, including human experimentation data. It is the primary evidence of Japanese biological warfare, and proves close institutional and personnel integration between the Army Medical School and Unit 731. Imperial Japanese Army Medical School, *Fifty-Year History of the Army Medical School* (Tokyo: Imperial Japanese Army, 1936); Imperial Japanese Army Medical Corps., *The Journal of the Army Medical Corps* [Periodical] (Tokyo: Medical Affairs Bureau, Japanese War Ministry, 1909–1945); Nagata Tetsuzan Publication Society, *Secret Records – Nagata Tetsuzan* (Tokyo: Fuyo Shobo Publishing,1972); JGSDF Medical School, *Hygienic History of Army in the Great East Asian War* (Tokyo: JGSDF Medical School, 1969–1971); Epidemic Prevention Research Laboratory of Army Medical School, *Epidemic Prevention Research Report of the Army Medical School* (Reprint Edition) **(**Tokyo: FujiShuppan Co., Ltd., 2004–2005).

32 Keiichi Tsuneishi, *Target Ishii: Espionage by Unit 731 and the U.S. Army* (Tokyo: Otsuki Shoten, 1984).

33 Concerning *Epidemic Prevention Research Report of the Army Medical School*, approximately 900 research reports have been discovered to date, constituting a significant body of historical materials formed during World War II. These reports, primarily published by members of the Army Medical School and Unit 731, cover topics such as biology, infectious diseases, epidemiology, and biological warfare.

34 Keiichi Tsuneishi made extensive use of wartime documents concerning Unit 731 and, for the first time, incorporated archives on Unit 731 preserved in the United States. This significantly broadened the scope of reference materials, thereby greatly enhancing both the empirical validity and academic credibility of his research findings.

35 Seiichi Morimura, *Akuma no Hōshoku* (The Devil’s Gluttony) (Banseisha, 1982).

36 Seiichi Morimura, *Shi-ningen no Shōmei* (New Proof of Man, Kadokawa Shoten, 1982).

37 Seiichi Morimura (ed.), *Saba kareta Nana-san-ichi Butai* (Unit 731 on Trial) (Banseisha, 1990).

38 Takashi Shimamura, Sanzennin no Seitai Jikkken: Nihon Gun “Saikin Butai” no Zaigo (3,000 Human Experiments: The Crimes of the Japanese Army’s Germ Warfare Unit) (Hara Shobo, 1981).

39 Shinsaku Ishida, *Akuma no Nihon Guni* (The Devilish Japanese Military Doctor) (Yamade Shobo, 1982).

40 Yoshiaki Hiyama, *Saikinsen Butai no Ishi wo Oe* (Pursuit of the Biological Warfare Unit Doctor) (Kodansha, 1980).

41 Ryoichi Naito, *Ro SL no Sōon: Ryoichi Naito Zatsubunshū* (The Noise of Old Steam Locomotives: A Collection of Ryoichi Naito’s Miscellaneous Writings) (Midorijuji (Privately Published), 1980).

42 Natsuko Yoshikai, *Kesenai Kioku; Yuasa Guni Seitai Kaibo no Kiroku* (Unforgettable Memory: A Record of the Vivisection of Dr. Yuasa, a Military Doctor) (Nicchu Shuppan, 1981).

43 Yoko Gunji, *Shōgen: Nanasanichi Ishii Butai* (Testimony: Unit 731 and the Ishii Unit) (Tokuma Shoten, 1982).

44 Yoko Gunji, *Shinsō: Ishii Saikinsen Butai* (Uncovering the Truth: Ishii’s Germ Warfare Unit) (Tokuma Shoten, 1982).

45 Sueo Akimoto, *I no Ronri wo Tou: Dai Nanasanichi Butai de no Taiken kara* (Questioning the Logic of Medicine: Based on Experiences in Unit 731) (Keisoshobo, 1983).

46 Sadao Koshi, *Hinomaru wa Akai Namida ni: Dai Nanasanichi Butaiin Kokuhakuki* (The Rising Sun is Dyed Red with Tears: Confessions of a Member of Unit 731) (Kyōiku Shiryō Shuppankai, 1983).

47 Hisato Yoshimura, *Kijū Kaiko* (Looking Back on My 77th Year), privately published, 1984. Hisato Yoshimura, former leader of the frostbite research unit in Unit 731, became president of Kyoto Prefectural University of Medicine after the war. He wrote this memoir at age 77. As the recollections of a core member of Unit 731, it holds significant research value. In this work, he refutes Morimura Seiichi’s *The Devil’s Gluttony*, arguing that conducting frostbite experiments stemmed from superior orders and was an unavoidable passive act. Yoshimura repeatedly attempts to legitimise these medical crimes, offering no reflection or remorse. Instead, he frequently boasts about his wartime academic achievements in frostbite research. As he wrote on page 183 of the book: “My research findings during the war (referring to experiments on human frostbite) were published in English-language physiology journals after the war, sparking significant interest among scholars in Europe and America. Even today, the experimental methods that advanced this research continue to serve as a reference not only in Japan but also for experiments conducted at universities and research institutes worldwide.”

48 Masao Takezawa, *Tōgō Kaishi* (Tōgō Society Journal), privately published, 1986.

49 Nanasanichi Kenkyūkai (731 Research Society), *Saikinsen Butai* (Biological Warfare Unit), Banseisha, 1996.

50

Robert Harris and Jeremy Paxman , A Higher Form of Killing: The Secret Story of Chemical and Biological Warfare (Hill&Wang, 1982).

51 David Charles Rea (dir.), *Unit 731: Did the Emperor Know?*, Television South (TVS), 13 August 1985. Available at https://collections-search.bfi.org.uk/web/Details/ChoiceFilmWorks/150299115.

52

Peter Williams and David Wallace , Unit 731: Japan’s Secret Biological Warfare in World War II (London: Hodder and Stroughton and New York: The Free Press, 1989).

53 This work is the first English-language book on the subject of Unit 731, possessing groundbreaking significance. Consequently, relevant studies by Western scholars, such as *Hidden Atrocities: Japanese Germ Warfare and American Obstruction of Justice at the Tokyo Trial*, as well as degree theses like `Biological Warfare: the Battlefield of the Future’ (Shauna L. Doolittle, Thesis for the Degree of Master of Science in Nursing, Gonzaga University, 2000), are all unable to bypass this seminal text in their literature reviews and citations.

54 John Powell, ‘A Hidden Chapter in History’, *Bulletin of the Atomic Scientists*, 37, 8, 1981.

55 Sheldon Harris, ‘Japanese Biological Warfare Experiments and Other Atrocities in Manchuria,1932–1945, and the Subsequent United States Cover Up: a Preliminary Assessment’, *Crime, Law and Social Change*, 15, 3, 1991.

56

Sheldon Harris , Factories of Death: Japanese Biological Warfare, 1932–45, and the American Cover-up (New York: Routledge, 1994).

57 Sheldon Harris, *Factories of Death* (Japanese Edition), S. Kondo (trans.) (Tokyo: Kashiwa Shobo,1999); Sheldon Harris, *Factories of Death* (Chinese Edition), X. Wang (trans.) (Shanghai: Shanghai People’s Publishing House, 1999).

58 Walter Eugene Grunden, ‘Science under the Rising Sun: Weapons Development and the Organization of Scientific Research in World War II Japan’, Dissertation for the Degree of Doctor of Philosophy, University of California Santa Barbara, 1998.

59 Masakatsu Ota, *Nanasanichi Menzeki no Keifu: Saikinsen butai to Hisō no Fairu* (Unit 731’s Immunity: Secret Files of the Biological Warfare Unit) (Nihon Hyōronsha, 1999).

60 Saburo Ienaga, born in 1914, was a contributor to the Japanese high school history textbook *New History of Japan.* This edition of the textbook was noted for its relatively objective account of historical facts and was widely adopted by Japanese high schools in the postwar period.

61 Third Petty Bench of the Supreme Court of Japan, *Supreme Court Cases, Civil Cases* (Minshū), 51, 7, 2921, available at https://www.courts.go.jp/app/hanrei_jp/detail2?id=52529, accessed 31 December 2025.

62

Yoshiko Nozaki , War Memory, nationalism and Education in Postwar Japan, 1945–2007: The Japanese History Textbook Controversy and Ienaga Saburo’s Court Challenges (London: Routledge, 2008).

63 Seiichi Morimura, *Unit 731 on Trial* (Tokyo: Banseisha, 1990).

64
*Ibid*.

65 Hata Ikuhiko holds a Doctor of Laws (LL.D.) from the University of Tokyo and serves as a professor at Takushoku University in Japan. Eguchi Keiichi, Doctor of Literature from Kyoto University, Professor at Aichi University of Japan, and a distinguished authoritative scholar in modern Japanese history.

66 Akira Tanaka and Takao Matsumura, *Nanasanichi Butai Sakusei* (Documents Created by Unit 731), Fuji Shuppan, 1996. In this book, the author discovered for the first time two medical experiment reports from Unit 731 within Japanese folk archives: one detailing chemical weapon human experimentation, and another concerning tetanus human experimentation. These two human experiment reports, written in Japanese, constitute the most direct evidence of criminal activity. These newly uncovered materials play a pivotal role in constructing the historical chain of evidence. From this point forward, denying the medical crimes committed by Unit 731 has lost its academic legitimacy.

67 Takao Matsumura, *Arguments for Unit 731* (Tokyo: Banseisha, 1994). Pages 64 to 69 of this book detailed the public response following the declassification of Documents Created by Unit 731. The content was divided into two sections: Reactions in Japan and Reactions Abroad. It included reports from Japan’s *Mainichi Shimbun*, China’s *People’s Daily*, and the UK’s *The Times, Daily Mail*, and *The Daily Telegraph.*

68 731 Biological Warfare Trial Campaign Committee, ABC Planning Committee and Unit 731 Biological Warfare Victims State Compensation Claim Litigation Defense Team, *Biological Warfare on Trial* (Vol. 1–8) (Tokyo: Internally Published Document, 2002).

69 Tokyo District Court, Heisei 14 [2002] Case Nos. Wa 16684 and Wa 27579, judgment dated 27 August 2002, Japanese Court Judgments, available at https://www.courts.go.jp/hanrei/5795/detail4/index.html.

70 Yuanming Zhang (trans), `Significance of the Court Decision of First Instance on the Lawsuit against Japan’s Implementing the Bacteriological Warfare in China and Tasks to Be Fulfilled in Second Instance’, *Journal of Changde Teachers University (Social Science Edition)*, 1 (2003), 3-9.

71 These academic communities include, on the Chinese side: the Harbin Academy of Social Sciences/Unit 731 Issue International Research Center, Unit 731 War Crimes Exhibition Hall, Zhejiang Yiwu Research Center for Bacteriological Warfare Issues, National Library of China, and on the Japanese side: NPO 731 Unit/Bacteriological Warfare Resource Center, Meiji Gakuin University, Institute for International Peace Studies, and the Japanese bar association & civic groups. 731 Biological Warfare Trial Campaign Committee, ABC Planning Committee, Unit 731 Biological Warfare Victims State Compensation Claim Litigation Defense Team, *Biological Warfare on Trial*, (Vol. 1–8), Tokyo: Internally Published Document, 2002.

72 Takeshi Yoshinaka, a graduate of the Graduate School of Medicine, Kyoto University, edited *Unit 731 and Universities*. Shozo Azami, an alumnus of the Faculty of Medicine, Kanazawa University, authored *War and Medicine* Tetsuro Kato, a graduate of the Faculty of Law, the University of Tokyo, published *Unit 731 and Post-war Japan*. Additionally, Keishiro Karita, an alumnus of Tohoku University, serves as a professor of physiology at his alma mater. Katsuo Nishiyama, a graduate of Kyoto University, works as a professor of preventive medicine at Shiga University of Medical Science. Keiko Suenaga, also from Tohoku University, is an associate professor at Fukushima Medical University.

73 Waichiro Omata, *Kenshō Jintai Jikken: Nanasanichi Butai Nachi Igaku* (Verification of Human Experimentation: Unit 731 and Nazi Medicine) (Dai San Bunmeisha, 2003).

74 Katsuo Nishiyama, *Sensō to Igaku* (War and Medicine) (Bunrigaku, 2013).

75 Fuyuko Nishizato, *Seibutsusen Butai Nanasanichi: Amerika ga Menzai Shita Nihongun no Sensō Hanzai* (Unit 731: The Japanese Army’s Biological Warfare Unit That America Pardoned) (Kusanone Shuppankai, 2002).

76 Fukiko Aoki, *Nanasanichi: Ishii Shirō to Saikinsen Butai no Yami wo Abaku* (731: Unveiling the Darkness of Unit 731 and Shiro Ishii) (Shinchosha, 2008).

77 Jingbao Nie, Nanyan Guo, et al., *Japan’s Wartime Medical Atrocities: Comparative Inquiries in Science, History, and Ethics* (New York: Routledge, 2010).

78 Jeanne Guillemin, *Hidden Atrocities: Japanese Germ Warfare and American Obstruction of Justice at the Tokyo Trial* (New York: Columbia University Press, 2017).

79

Kenneth L. Port , Deciphering the History of Japanese War Atrocities: The Story of Doctor and General Shiro Ishii (Durham: Carolina Academic Press, 2014).

80 Shoji Kondo, *Nanasanichi Butai Saikinsen Shiryō Syūsei* (A Collection of Documents on Unit 731’s Biological Warfare) (Kashiwa Shobo, 2003).

81 Epidemic Prevention Research Laboratory, Army Medical College, *Rikugun Guni Gakukō Bōeki Kenkyū Hōkoku (Dai Ni Bu)* (Report on Epidemic Prevention Research at the Army Medical School (Part 2) (Reprint)), Fuji Shuppan, 2004-2005.

82 Omata, born in 1950, earned his Doctor of Medical Science degree from the Faculty of Medicine, Nagoya City University in 1980. He once served as Director of Ueno Psychiatric Clinic in Tokyo and Director of the Tokyo Association of Insurance Physicians. Grounded in a medical practitioner’s observational perspective, this book presented the first comprehensive medical historical examination comparing Unit 731 with Nazi medical atrocities, endowing it with remarkable pioneering significance. Waichiro Omata, *Kenshō Jintai Jikken: Nanasanichi Butai Nachi Igaku* (Verification of Human Experimentation: Unit 731 and Nazi Medicine) (Dai San Bunmeisha, 2003).

83 Statistics were compiled based on thematic papers and reports published in the *Journal of 15-Years War and Japanese Medical Science and Service* founded in 2000 and its successor publication, the journal *War and Medicine*.

84 Japanese scholar Waichiro Omata offered a representative comparative analysis of wartime medical crimes committed by Japanese and German military physicians. In Verification of Human Experimentation: Unit 731 and Nazi Medicine. Omata systematically examined the similarities and differences between Japanese and German medical atrocities throughout prewar, wartime, and postwar periods.

85 Shozo Azami, ‘“The Medical Crime in the Fifteen Years’ War” and Our Task Today’, *Journal of 15-Years War and Japanese Medical Science and Service*, 8, 2 (2008), 34-36.

86 Katsuo Nishiyama, *Sensō to Igaku* (War and Medicine) (Bunrikaku, 2013).

87 The Research Society for Fifteen Years War and Japanese Medical Science and Service, *NO MORE 731: Nihongun Saikinsen Butai: Igakusha Ishi Tachi no Ryōshin wo Kaketa Kyūmei* (NO MORE 731: The Japanese Army’s Biological Warfare Unit – A Conscientious Investigation by Medical Professionals), Bunrikaku, 2015. This volume, edited by renowned scholar Katsuo Nishiyama to mark the 70th anniversary of the Second World War’s conclusion, is a collection of essays primarily authored by Japanese scholars. Focusing on medical history and bioethics, it includes papers by seven medical experts, investigative reports by two journalists, an article by Yoshio Shinozuka – a former child soldier of Unit 731 – an article by an architectural scholar, and Yang Yanjun’s ‘Investigation and Research on the Site of Unit 731’.

88 The Research Society for Fifteen Years War and Japanese Medical Science and Service, *Sensō, Nanasanichi to Daigaku, Igaku Daigaku* (*War and Unit 731, Universities and Medical University*) (Tokyo: Bunrikaku, 2016).

89 Tsuneishi Keiichi, *Nanasanichi Butai Zenshi: Ishii Kikan to Gungaku Kansan Kyōdōtai* (Complete History of Unit 731: The Ishii Institution and the Military-Academic-Industrial-Government Complex) (Tokyo: Koubunken, 2022).

90 Takeshi Yoshinaka, Nanasanichi Butai to Daigaku (Unit 731 and the University) (Kyoto: Kyoto University Press, 2022).

91

Jingbao Nie , ‘The United States Cover-up of Japanese Wartime Medical Atrocities: Complicity Committed in the National Interest and Two Proposals for Contemporary Action’, The American Journal of Bioethics, 6, 3, 2006 10.1080/1526516060068635616754432.

92 Jing-Bao Nie, ‘The United States Cover-Up of Japanese Wartime Medical Atrocities: Complicity Committed in the National Interest and Two Proposals for Contemporary Action’, *The American Journal of Bioethics*, 6, 3 (2006), 21–33.

93 Katrien Devolder, ‘U.S. Complicity and Japan’s Wartime Medical Atrocities: Time for a Response’, *The American Journal of Bioethics*, 15, 6 (2015), 40–49.

94 Julie Aultman, ‘When Saying Sorry Is Not Enough: Acknowledging Past Wrongs in Human Subjects Research’, *The American Journal of Bioethics*, 15, 6 (2015), 57–59.

95

Kenneth L. Port , Deciphering the History of Japanese War Atrocities: The Story of Doctor and General Shiro Ishii (Durham: Carolina Academic Press, 2014).

96

Jeanne Guillemin , Hidden Atrocities: Japanese Germ Warfare and American Obstruction of Justice at the Tokyo Trial (New York: Columbia University Press, 2017).

97

Kishor Johnson , ‘A Scientific Method to the Madness of Unit 731’s Human Experimentation and Biological Warfare Program’, Journal of the History of Medicine and Allied Sciences, 77, 1, 2022 10.1093/jhmas/jrab04434897467.

98 Valerie J. Cranmer, ‘In Judgment of Unit 731: A Comparative Study of Medical War Crimes Trials after World War II’, *The Journal of American-East Asian Relations*, 30, 1 (2023), 32–60.

99
*Ibid.*

100
*Ibid.*, 54.

101 Central Archives, Rìběn qīnhuá zhànfàn bǐgōng xuǎnbian (Selected Confessions of Japanese War Criminals Who Invaded China) (Beijing: Zhonghua Book, 2015).

102 Chengmin Jin and Yanjun Yang (eds), *Factual Record of the Crimes of the Unit 731 of the Japanese Army in Invasion of China (60 Volumes)* (Beijing: China Peace Publishing House, 2025).

103 Zhiyuan Chen, Qīnhuá Rìjūn Chángdé xìjūn zhàn yánjiū cóngshū (A Series of Studies on the Biological Warfare Waged by the Japanese Invaders in Changde) (Beijing: China Social Sciences Press, 2015).

104 Chenqi Li, *Qīnhuá Rìjūn Qībǎisānshīyībùduì Jiùzhǐ Xìjūn Shíyànshì jí Tèshè Jiānyù Yízhǐ Kǎogǔ Fājué Bàogào* (Archaeological Excavation Report of the Former Site of the Bacterial Laboratory and Special Prison of the Japanese Invading Army’s Unit 731) (Beijing: Science Press, 2018).

105 Yanjun Yang, *Guāndōngjūn Fángyì Gěishuǐbù Liúshǒu Míngbù* (Namelist of the Kwantung Army’s Epidemic Prevention and Water Supply Department) (Beijing: Central Compilation & Translation Press, 2021).

106 Yanjun Yang, *Jiadi Yibawuwu Budui Liushou Mingbu* (Namelist of the Unit 1855) (Beijing: Central Compilation & Translation Press, 2021).

107 Yanjun Yang, *Nanfangjun Fangyi Geshuibubu Liúshǒu Míngbù* (Namelist of the Southern Army’s Epidemic Prevention and Water Supply Department) (Beijing: Central Compilation & Translation Press, 2022).

108 Zhiyuan Chen, *Riben Qinhua Xijun Zhan* (Japan’s Biological Warfare Against China) (Beijing: China Social Sciences Press, 2014).

109 Zhonghou Xie, *Riben Qinhua Xijun Zhan Zuixing Diaoyan Baogao* (Research Report on the Crimes of Japanese Biological Warfare Against China) (Beijing: CCP Party History Press, 2010).

110 Jianxin Zhu, *Shengwu Zhanshi* (A History of Biological Warfare) (Beijing: Times Publishing House, 2015).

111 Xiliang Wang, ‘The Main Content, Historical Value, and Significance of Newly Discovered Documents on Japanese Biological Warfare’, *Research on the Anti-Japanese War*, 1, 2012.

112 Jian Gao, ‘Unit 731 and the Allied POW Camp in Shenyang’, *Studies in the History of the Anti-Japanese War*, 2, 2012.

113 Yanjun Yang, ‘Examining the Personnel Composition of Unit 731’, *Historical Research*, 3, 2019.

114 Yanjun Yang and Yuehim Tam, *Unit 731: Laboratory of the Devil, Auschwitz of the East* (Stroud: Fonthill Media, 2018).

115 As noted in Zhonghou Xie, *Research Report on the Crimes of Japanese Bacteriological Warfare during the Invasion of China* (Beijing: Party History Press of the Communist Party of China, 2010), 182: ‘During the Japanese war of aggression against China, biological warfare was implemented… Preliminary estimates suggest that there were approximately 7 million infected patients, of whom about 2 million died’. It is evident that this figure lacks support from scientific data.

116 Yi-Jong Suh, ‘Bare Life in Manchuria and the Victims of Human Experiments in the Special Prison of Unit 731’, *Manchurian Studies* (Korean), 18, 2014.

117 Kyu-Hwan Sihn, ‘The Memory of Biological Warfare and New Directions for Patriotic Education Base Construction in China’, *Shi Lin* (Korean), 47, 2014.

118 Alexander V. Alepko, ‘The Khabarovsk War Crimes Trial of 1949 and the Cruel Human Experiments of Japan’s Unit 731’, *Far Eastern Culture and Science* (Russian), 1, 2019.

119 The Research Society for Fifteen Years War and Japanese Medical Science and Service renamed in 2020 as Research Society for War and Medicine.

## Supplementary material

The supplementary material for this article can be found at http://doi.org/10.1017/mdh.2026.10062.

