# Supplementary material for: Global research on Unit 731 medical atrocities: from the 1950s to the 2020s
Source: Med Hist. 2026 Oct;70(4):651–71. doi: 10.1017/mdh.2026.10062 (PMC13735525; doi:10.1017/mdh.2026.10062)
Supplement: Supplementary file 1 [file S0025727326100623sup001.pdf]

Survey of Doctoral Degree Holders Among Unit 731 Members

The timing of doctoral degree attainment among former members of Unit 731 can be categorized into two types. The first type includes those who obtained their doctoral degrees before August 15, 1945 (serial numbers 1–75). Among them, some joined Unit 731 after earning their degrees, while others completed their doctoral studies while serving in the unit. The second type comprises those who were awarded doctoral degrees after August 15, 1945 (serial numbers 76–139). Most of these individuals leveraged academic achievements gained during their tenure in Unit 731 to apply for their doctoral degrees. For details, please refer to the table below.

Survey of Doctoral Degree Holders among Unit 731 Members[①]

order	surname and personal name	PhD dissertation topic	degree-granting body	Time of degree conferral	Degree Level	Rank and Military Grade
1	Watanabe Bian	Changes in bacterial volume during lysis phenomenon	Keio Imperial University	May 12,1925	medical science	artificer
2	Anton Hongji	Biological Studies on Chain-Like Coccus	Keio University	October 7, 1925	medical science	artificer
3	Kitano Masaji	Experimental Study on Serum Resistance to Typhoid and Paratyphoid Bacteria	Tokyo Imperial University	November 25,1926	medical science	lieutenant general
4	Ishii Shiro	Research on Gram-positive diplococci	Keio Imperial University	June 24,1927	medical science	lieutenant general
5	Ikuchi Qi	Regarding the relationship between Pseudomonas aeruginosa and Clostridium splenicum, as well as studies on Pseudomonas aeruginosa-like pathogens	Tokyo Imperial University	December 11,1928	medical science	major general
6	Er Yu De San	Chemical Studies on Sandalwood Oil and a Novel Crystalline Sesquiterpene Alkohol	Keio University	December 15,1928	medical science	artificer
7	Shibata Tō	On Intestinal Secretion in Insects	Keio Imperial University	March 15,1929	Neo-Confucianism	artificer
8	Sato Shunji	Experimental Study on Amyloidosis in Rabbits	Osaka Imperial University	September 20,1930	medical science	major general
9	Imakin Kōkan	Experimental Study on the Evaluation of Hepatic Glycogen Synthesis Capacity During Artificial Fever Induction Through	Tokyo Imperial University	March 19,1931	medical science	major general

Compiled from materials at the National Diet Library of Japan.

		Perfusion Tests (German)				
10	Yamashita Ryōyu	Research on Syphilis Reactions	Osaka Imperial University	April 27,1931	medical science	major general
11	The remaining funds are stored in the treasury.	Experimental Study on In Vivo Staining of Bone and Teeth	Keio Imperial University	September 3, 1931	medical science	major general
12	Tadaaki Masamune	Research on the Number of Precipitate Particles in the Settlement Reaction	Keio Imperial University	September 3, 1931	medical science	Colonel
13	Tianchen Liangdao	Contact Reduction of Resorcinol and Its Derivatives under High Temperature and Pressure Conditions	Keio Imperial University	March 14,1932	Neo-Confucianism	Adjuvant in pharmaceutical preparations
14	Nishikuni Hikaru	Effects of irritant gases on the circulatory system	Keio Imperial University	November 11,1932	medical science	Colonel
15	Yonshan Tarō	Experimental Studies on Non-Perceptual Evaporation and Respiratory Gas Metabolism	Oyama Medical University	November 14,1932	medical science	Colonel
16	Kurana Kihiko	Research on Plague	Keio University	December 16,1932	medical science	artificer
17	Kato Shinichi	Efficacy of various hormonal preparations from the perspective of cardiac function	Nagoya Medical University	February 3, 1933	medical science	Colonel
18	Yoshimichi Kobayashi	Effect of Triphenylmethane Pigment on Spleen Ankylosis Bacteria	Keio University	April 11,1933	medical science	first lieutenant
19	Takashi Murakami	Supplementary information on lead poisoning in experiments: Specifically regarding the effects of colloidal solution and suspension injection, as well as splenectomy, on lead poisoning	Kumamoto Medical University	September 28,1933	medical science	Colonel

20	Ishii Yao	The effect of asphyxia on pancreatic juice secretion	Osaka Imperial University	August 10,1933	medical science	artificer
21	Taro Yuan Tian	Prevalence of Schistosomiasis japonica infection and the impact of jaundice on development	Keio Imperial University	November 21,1933	medical science	Colonel
22	Matsuzaki Yang	Iris-adrenaline hypersensitivity reactions are common following superior cervical ganglionectomy.	Keio University	May 11,1934	medical science	major general
23	Watanabe Shiro	Changes in the phagocytic function of subcutaneous tissue cells in various experimental diseases	Kanazawa Medical University	May 21,1934	medical science	not quite clear
24	Kasahara Shiro	On Tissue Culture of the Adrenal Gland	Keio University	March 6, 1935	medical science	artificer
25	Antongqing	The immunogenic efficacy of inactivated Salmonella typhimurium vaccine against murine infection by the bacterium	Keio University	April 5, 1935	medical science	first lieutenant
26	Kawashima Kiyoshi	Research on Fevre's pseudotuberculosis bacillus	Chiba Medical University	April 16,1935	medical science	major general
27	Yasuzo Okamoto	Supplementary Studies on the Pathological Anatomy and Histology of the Hypophysis, Particularly Its Anterior Lobe	Keio University	May 11,1935	medical science	artificer
28	Yoshimura Hisato	Study on the Determination of Blood Hydrogen Ion Concentration Using the Glass Electrode Method (English)	Keio Imperial University	April 7, 1936	medical science	artificer
29	Taniideiwa	Experimental Studies on Oral Immunization Against Typhoid and Paratyphoid Bacteria	Keio Imperial University	May 4, 1936	medical science	artificer
30	Mako Kenji	Research on Vibrio cholerae and similar vibrios	Kyushu Imperial University	December 24,1936	medical science	artificer
31	Ryoichi Naito	Supplementary Notes on Phage Research	Keio Imperial University	April 8, 1937	medical science	Lieutenant Colonel
32	Black and White	Research on the serotypes of Gertnerella enterocolitica	Hokkaido Imperial University	July 26,1937	medical	Lieutenan

	Show Outside				science	t Colonel
33	Saito Yukichirō	Doubts Regarding the Existence of Redox Potential in the Hemoglobin·Metohemoglobin System (English)	Keio Imperial University	July 30,1937	medical science	artificer
34	Sasaki Yoshio	The presence of coliform bacteria in the urine of patients with acute peritonitis	Keio Imperial University	December 13,1937	medical science	Lieutenant Colonel
35	Hayakawa Kiyoshi	Relevant Studies on Variants of Salmonella Typhi	Tokyo Imperial University	December 23,1937	medical science	Lieutenant Colonel
36	Shi Guangxun	Histological changes and incidence patterns following viral inoculation of inguinal lymphogranuloma into the brains of mice to induce infection	Tokyo Imperial University	December 27,1937	medical science	artificer
37	Kaiyuan Qin	Cultivation Test for Streptococcus suis	Keio University	December 27,1937	medical science	artificer
38	Ando Keisaburo	Research on Fixed Virulence in Rabies	Tokyo Imperial University	January 15,1938	agronomy	artificer
39	Ishikawa Daidō Yūmaru	Research on Redox Voltages (German)	Keio Imperial University	March 3, 1938	medical science	artificer
40	Okamoto Kōsō	Organizing Research on Iron (German)	Keio Imperial University	March 3, 1938	medical science	artificer
41	Rin Ichiro	On the significance of pigments in the respiratory system, particularly pigment uptake granules (German)	Keio Imperial University	March 3, 1938	medical science	artificer
42	Tanioka Ryosuke	Research on Soil Nematodes	Tokyo Chihui-kai Medical University	March 24,1938	medical science	not quite clear
43	Tanaka Eiji	Vascular Distribution and Pigmentation in the Ctenoid Membrane of Japanese Birds (English)	Keio Imperial University	April 23,1938	medical science	Technical Lieutenant Colonel
44	Iike Tadafumi	Serological chemical reaction to rat leishmaniasis (M.H.H. reaction)	Kyushu Imperial University	July 29,1938	medical science	Colonel
45	Niji Hideyoshi	Histological examination of brain and spinal cord tissues	Kanazawa Medical University	November 18,1938	medical	artificer

		in rabbits with neurosyphilis			science	
46	Yasukawa Takashi	Research on Colibacterium spp. in Fish	Tokyo Imperial University	December 1, 1938	agronomy	artificer
47	Kubao Institute Akio	Research on 痘苗病毒 culture	Kyoto Prefectural Medical College university	February 14,1939	medical science	artificer
48	Old Tan Masumi	Retrieval of Shigella carotis carriers in Nagoya City	Nagoya Imperial University	September 6, 1939	medical science	artificer
49	Spring Day: Loyalty and Kindness	Study on the Specificity of the Plague Bacterium Sedimentation Reaction	Keio University	March 4, 1940	medical science	artificer
50	Hōjō has succeeded.	Study on the Variation of Vibrio cholerae on Acidic and Alkaline Media	Tokyo Imperial University	October 28,1940	medical science	Colonel
51	Hamada Toshihiro	Research on the Mycorrhizal Association of Rhodopseudomonas erythrosphaera	Keio Imperial University	December 4, 1940	Neo-Confucianism	artificer
52	Matsuda Masanobu	Cultivation of bacteria and trypanosomes on plasma culture medium	Keio Imperial University	December 7, 1940	medical science	artificer
53	Yanaguchi Hachiro	Morphological Study on Liver Cancer Progression Using Methyl Yellow	Osaka Imperial University	April 7, 1941	medical science	second lieutenant
54	Mitsuhiko Takahashi	Clinical Studies on Surface Tension of Human Body Fluids	Keio Imperial University	May 19,1941	medical science	artificer
55	Mori and Yuu	The Effect of Organ Feeding on the Development of Experimental Hepatocellular Carcinoma	Osaka Imperial University	November 27,1941	medical science	artificer
56	Kondo Junichiro	Intraocular pressure measurement and intraocular pressure curve tracing device	Capital Imperial University	November 27,1941	medical science	artificer
57	Iino Kazuo	Continuous dosing experiments with various inorganic iron compounds	Manchurian Medical University	December 3, 1941	medical science	artificer

58	Soanfu	Pathological Histological Studies of Primary Tumors of the Central Nervous System, Particularly Gliomas	Tokyo Imperial University	January 30,1942	medical science	artificer
59	Li Qiu Yao	Research on the Correlation Between Vascular Ion Concentration in Rabbits	Capital Imperial University	February 16,1942	medical science	artificer
60	Ma Shanju San	Research on Complement Function	Tokyo Imperial University	June 18,1942	medical science	not quite clear
61	Miyagawa Masahiro	On the Issue of X-ray Irradiation Dosage Based on Autopsy Findings of Esophageal Cancer Patients Undergoing Rotational X-ray Irradiation	Tokyo Imperial University	December 1, 1942	medical science	artificer
62	Suzuki Keiji	Research on the Dried Preservation of Live Bacteria for Special Mass Production: Using 炭疽杆菌, Typhoid 杆菌, and Cholera 杆菌 as Experimental Subjects	Keio Imperial University	February 6, 1943	medical science	Lieutenant Colonel
63	Yamura Takashi	Understanding of Septicemic Vibrio	Kanazawa Medical University	April 13,1943	medical science	Major
64	Takehara Takashi	Experimental Study on Endocrine Disorders and Blood Pressure	Keio Imperial University	May 4, 1943	medical science	artificer
65	Yoshikawa Kazutoshi	Research on the Synthesis of Ox烷ylbenzones	Tokyo Imperial University	May 4, 1943	pharmacy	Captain of the Chemical Corps
66	Tanaka Toyoshi	A variant strain of the pseudotuberculosis bacterium in rodents	Keio University	July 1, 1943	medical science	Major
67	Ota Sumi	Experimental Study on the Biological Characteristics of Clostridium splenicum	Oyama Medical University	July 27,1943	medical science	Colonel
68	Nishimura Eiji	Experimental Studies on Shigella spp.	Oyama Medical University	July 27,1943	medical science	Colonel

69	Kanazawa Kenichi	On the Culture of Rabies Virus	Tokyo Imperial University	October 12,1943	medical science	artificer
70	Akimoto Hisahideo	A Serological Re-examination of Hemoglobin	Tokyo Imperial University	February 22,1944	medical science	artificer
71	Panasonic Motoki	Changes in blood and tissue profiles during artificial fever induction	Tokyo Imperial University	April 7, 1944	medical science	Major
72	Takahashi Masahiko	Research on "Yersinia pestis" and "phages"	Keio University	April 13,1944	medical science	Major
73	Fujimura Yoshinosuke	Vitamin C Research	Keio Imperial University	February 15,1945	agronomy	artificer
74	Keiichi Nom野	Study on the preservation method of live attenuated plague bacteria using vacuum freeze-drying	Keio Imperial University	April 13,1945	medical science	Major
75	Manabu Hayano	Comparative Determination of Various Typhoid Antigens	Manchurian Medical University	July 7, 1945	medical science	artificer
76	Hirazawa Masahide	Experimental Study on the Plague Vector Capacity of Canine Fleas and Canine Combs	Keio Imperial University	September 26,1945	medical science	Lieutenant Colonel
77	Tadashi Koguchi	Classification of the Enterobacteriaceae family	Kumamoto Medical University	May 17,1946	medical science	Major
78	Mitsumoto Tsunehiko	Applications of Ethylene Glycol Derivatives in Microbiology	Keio Imperial University	July 29,1946	medical science	Major
79	Furinari Kishi Shishin	Research on scrub typhus	Niigata Medical University	November 2, 1946	medical science	Major
80	Watanabe Ken	On the Preventive Efficacy of Tetanus Toxin	Kumamoto Medical University	November 14,1946	medical science	Colonel
81	Nakano Nobuo	Research on the Intra-Organ Plague Bacterium Staining Method	Keio University	November 28,1946	medical science	Major

82	Amamiya Miki	Characteristics of High-Pressure Co-culture of Yersinia pestis with Glucose and Isotonic Solution	Keio University	November 28,1946	medical science	Major
83	Hidetoshi Higuchi	Research on the Culture of Wild Rabbits Pathogens	Keio University	February 17,1947	medical science	Major
84	Takagawa Nobuyasu	Investigative Study on the Infection Status of Tropical Strychniasis in Java	Tokyo Chiho-kai Medical Department university	April 10,1947	medical science	Major
85	Fujimoto Taro	Relevant research on Shigella	Kanazawa Medical University	July 18,1947	medical science	artificer
86	Tanaka Junyu	Research on Antimicrobial Substances Produced by Domestic Actinomycetes	Kyoto University	February 19,1948	medical science	Major
87	Kaneko Junichi	Basic Investigation on Rainfall Distribution	Tokyo University	January 10,1949	medical science	Major
88	Watanabe Makoto	Hematological findings and clinical manifestations in patients with rickets symptoms suspected to be caused by rickets or malnutrition	Nagoya University	January 10,1949	medical science	senior captain
89	Saimou Saizumaru	Two or three biological studies on iron	Kyoto University	January 10,1949	medical science	artificer
90	Takahashi Biography	Immunological Studies on Major Infectious Diseases in Northern Asia	Northeastern University	April 1, 1949	medical science	Lieutenan t Colonel
91	Miyazaki Junjin	Research on Active Immunization Against Tetanus and Gas Gangrene	Kyoto University	April 4, 1949	medical science	Major
92	Otsu Takejiro	Immunological Supplement to Red Dysentery and Pediatric Red Dysentery	Keio University	May 14,1949	medical science	first lieutenant
93	Hideo Nagamoto	Research on Non-Specific Serum Reactions	Kyoto Prefectural Medical College university	August 4, 1949	medical science	not quite clear
94	Iida Toshikō	Research on Cold Blood Vessel Response	Kyoto Prefectural Medical	August 4, 1949	medical	artificer

			University		science	
95	The Three Retaining Light Men	Experimental Study on the Efficacy Evaluation of Chloramine as a Bactericidal and Disinfectant Agent	Tokyo University	January 28,1950	medical science	artificer
96	Fujihara Ruzō	Research on Typhoid Bacillus Antigenic Substances	Northeastern University	March 31,1950	medical science	artificer
97	Suzuki Jiro	Chest Sympathetic Nerve Surgery and the "Time Value"	Nagoya University	August 25,1950	medical science	artificer
98	Noyasaki Toshi	Research on Dermatitis Caused by Yellow Moths and Their Larvae	Tokyo Medical University	December 5, 1950	medical science	Major
99	Ekiou Fujie	Research on Enterococcus coli	Kyoto Prefectural Medical College university	March 12,1951	medical science	Lieutenant Colonel
100	Arai Iwao	Research on the Developmental Aspects of Salmonella Typhi	Tokyo University	May 31,1951	pharmacy	Major of Pharmacy
101	The military officer who lowered the flag	Research on the exotoxins of Yersinia pestis	Tokyo University	January 25,1952	medical science	Major
102	Masajiro Honda	Research on Bone Marrow Findings in Diabetic Patients	Northeastern University	August 13,1952	medical science	Major
103	Asahina Masataro	Morphological Studies on the Ancient Dragonfly	Hokkaido University	April 4, 1953	Neo-Confucianism	artificer
104	Idle Xiubang	Research on Arterial Pulse Wave Correlation	Kyoto University	July 24,1953	medical science	first lieutenant
105	Ito Shizuya	Biochemical Study on the Utilization of Papaya Nectinase to Enhance Antibody Globulin Production in Test Tubes	Hokkaido University	August 19,1953	agronomy	artificer
106	Shinji Takegumi	Research on Rickettsia and Virus Cultivation	Tokyo University	May 20,1954	medical	artificer

					science	
107	Nishiji Tadashin	Mechanism of Action of Carbon Disulfide	Kyoto University	October 12,1954	medical science	Major
108	Nekatsu Masahiro	Research on the bacteriolytic polysaccharide-mediated hemolytic reaction specific to red blood cells	Tokyo University	December 17,1954	medical science	Major
109	Juryu Fumihiko	Effects of methyl androstenediol (MAD) and parotin on nitrogen metabolism in chicken fetuses	Niigata University	March 7, 1955	medical science	Major
110	Guo Xingchang Lai	Research on Subtype A	Tokyo Medical and Dental Hospital university	May 27,1955	medical science	senior captain
111	Toshihiro Iwasaki	II. Pharmacology of Triethylenediamine-Based Antihistamines	Northeastern University	June 25,1955	medical science	Major
112	Matsura Shigeru	The developmental status of cells is assessed by measuring the diameter of blood cells in peripheral blood and bone marrow.	Kyoto University	February 9, 1956	medical science	senior captain
113	Suzuki Toshiichi	Research on the Live Defense Response: The Relationship Between Adrenal Hormones and Tissue Glycolysis and Tissue Respiratory Processes	Keio University	June 8, 1956	medical science	artificer
114	Mizaki Yoichi	Histological and cytological changes in the kidneys induced by electrical stimulation of the autonomic center	Osaka University	June 20,1956	medical science	Lieutenan t Colonel
115	Iwasaki Takashi	Distribution of nucleic acids in nematodes	Kyoto University	July 25,1956	medical science	artificer
116	Watanabe Teiyo	Re-evaluation of Artificial Pneumothorax Technique	Keio University	August 9, 1956	medical science	senior captain
117	Ichiwara Shigede	On Female Sexual Function and Urinary 17-Ketosteroids: A Study Focusing Particularly on Their Distribution	Tokyo University	March 2, 1957	medical science	Major
118	Oki Maki Ichio	Pharmacological Studies on Pineal Material	Osaka Medical University	April 17,1957	medical	Major

					science	
119	Suzuki Shigehiko	Electromyographic Study of Unilateral Paralysis	Northeastern University	April 17,1957	medical science	Major
120	Mukamura Takeshi	Synthesis of p-nitrobenzoic acid and bromobenzoylacetone and related studies on their bioavailability	Kyoto University	November 12,1957	pharmacy	Major of Pharmacy
121	Tadahiro Yoshikawa	Detection of adenoseptica pathogens	Keio University	November 15,1957	medical science	not quite clear
122	Nishida Shigehide	Serological Immunological Study on Familial Clustering of Non-Asymptomatic Infections of Japanese Encephalitis	Osaka University	May 14,1958	medical science	Major
123	Ketada Tatsuyuki	Study on the Cytological and Cytochemical Changes in Submandibular Gland Cells of Mice After Injection of Scopolamine	Keio University	July 5, 1958	medical science	senior captain
124	Sato Yukio	Study of live cross-sectional sections: Observations of cross-sections of the orbit and surrounding organs	Nagoya University	March 20,1959	medical science	artificer
125	Er Yu Hong	Pharmacological Studies on Cerebral Circulatory Isolation	Kagoshima Prefectural University of Medicine	April 2, 1959	medical science	Major
126	Yamada Jiro	A gastritis examination primarily conducted using X-ray imaging	Keio University	June 6, 1959	medical science	artificer
127	Ito Masahiko	Epidemiological Study on the Outbreak of Red Dysentery Observed by the Military Forces Stationed in Katsuta, Ibaraki Prefecture	Showa Medical University	June 24,1959	medical science	Major
128	Yoshino Takamasa	Effects of Several Drugs on Koch's Phenomenon Induced by BCG Vaccine	Tokyo University	July 13,1959	medical science	senior captain
129	Guan Bofu	Cytological Studies of the genus Morinda	Kyoto University	September 18,1959	agronomy	artificer

130	Ishida Shōfu	Clinical Study on Manchurian Epidemic Hemorrhagic Fever	Niigata University	November 2, 1959	medical science	Lieutenant Colonel
131	Hikaru Sakahara	Research on Experimental Anemia Induced by Antibody-Induced Bone Marrow Serum	Okayama University	February 3, 1960	medical science	Major
132	Kyuohiko Kasai	Clinical Research on Chemical Prevention of Pediatric Tuberculosis Incidence	Chiba University	March 29,1960	medical science	artificer
133	Yasuo Masanori	Research on Streptococcus hemolyticus toxins	Tokyo University	March 31,1960	medical science	artificer
134	Yamamoto Genji	Research on Manchurian Forest tick-borne encephalitis	Keio University	May 23,1960	medical science	Major
135	Yuto Ito	Experimental Studies on Coronary Artery Occlusion	Japanese University	June 28,1960	medical science	Lieutenant Colonel
136	Shigeo Shinazaki	Research on the Blood Purification Effect of Shock in Rabbits with Hyperlipidemia	Tokyo Women's Medical College university	August 13,1960	medical science	artificer
137	Chiyono Junno	Research on Experimental Bone Tumors	Yokohama Municipal University	March 31,1961	medical science	senior captain
138	Yamani Toyohiko	Research on the Virulence Titer Determination Method for Cattle Pox Virus	Japanese University	March 29,1965	Veterinary Medicine	artificer
139	Fujio Shimizu	Study on the Variation of Liquid Refractive Index with Light Intensity	Tokyo University	January 23,1967	Neo-Confucianism	artificer
